# Landscape of Mitochondria Genome and Clinical Outcomes in Stage 1 Lung Adenocarcinoma

**DOI:** 10.3390/cancers12030755

**Published:** 2020-03-23

**Authors:** Lovely Raghav, Ya-Hsuan Chang, Yi-Chiung Hsu, Yu-Cheng Li, Chih-Yi Chen, Tsung-Ying Yang, Kun-Chieh Chen, Kuo-Hsuan Hsu, Jeng-Sen Tseng, Cheng-Yen Chuang, Mei-Hsuan Lee, Chih-Liang Wang, Huei-Wen Chen, Sung-Liang Yu, Sheng-Fang Su, Shin-Sheng Yuan, Jeremy J.W. Chen, Shinn-Ying Ho, Ker-Chau Li, Pan-Chyr Yang, Gee-Chen Chang, Hsuan-Yu Chen

**Affiliations:** 1 Institute of Statistical Science, Academia Sinica, Taipei 11529, Taiwan; lovey.bi03g@nctu.edu.tw (L.R.); d98548005@stat.sinica.edu.tw (Y.-H.C.); yuchengli@stat.sinica.edu.tw (Y.-C.L.); syuan@stat.sinica.edu.tw (S.-S.Y.); kcli@stat.sinica.edu.tw (K.-C.L.); 2 Institute of Bioinformatics and Systems Biology, National Chiao Tung University, Hsinchu 30010, Taiwan; syho@mail.nctu.edu.tw; 3 Bioinformatics Program, Taiwan International Graduate Program, Institute of Information Science, Academia Sinica, Taipei 11529, Taiwan; 4 Department of Biomedical Sciences and Engineering, National Central University, Taoyuan 32001, Taiwan; syic@ncu.edu.tw; 5 Institute of Medicine, Department of Surgery, Chung Shan Medical University Hospital, Taichung 40201, Taiwan; micc@www.cmuh.org.tw; 6 Faculty of Medicine, School of Medicine, National Yang-Ming University, Taipei 112, Taiwan; jonyin@gmail.com; 7 Division of Chest Medicine, Department of Internal Medicine, Taichung Veterans General Hospital, Taichung 40705, Taiwan; ckjohn@mail2000.com.tw (K.-C.C.); vghryan@gmail.com (K.-H.H.); tzeng64@yahoo.com.tw (J.-S.T.); 8 Institute of Biomedical Sciences, National Chung Hsing University, Taichung 402, Taiwan; jwchen@dragon.nchu.edu.tw; 9 Division of Thoracic Surgery, Department of Surgery, Taichung Veterans General Hospital, Taichung 40705, Taiwan; chuang5045@vghtc.gov.tw; 10 Institute of Clinical Medicine, National Yang-Ming University, Taipei 112, Taiwan; meihlee@ntu.edu.tw; 11 Department of Thoracic Medicine, Chang Gung Memorial Hospital, Tao-Yuan 33305, Taiwan; wang5726@gmail.com; 12 Graduate Institute of Toxicology, National Taiwan University, Taipei 10617, Taiwan; shwchen@ntu.edu.tw; 13 Department of Clinical Laboratory Sciences and Medical Biotechnology, College of Medicine, National Taiwan University, Taipei 10617, Taiwan; slyu@ntu.edu.tw; 14 Graduate Institute of Oncology, College of Medicine, National Taiwan University, Taipei 10055, Taiwan; shengfang@stat.sinica.edu.tw; 15 Department of Statistics, University of California Los Angeles, Los Angeles, CA 90095-1554, USA; 16 Center of Genomic Medicine, National Taiwan University, Taipei 10617, Taiwan; pcyang@ntu.edu.tw; 17 Department of Internal Medicine, National Taiwan University Hospital, Taipei 100, Taiwan; 18 Comprehensive Cancer Center, Taichung Veterans General Hospital, Taichung 40704, Taiwan; 19 Division of Pulmonary Medicine, Department of Internal Medicine, Chung Shan Medical University Hospital, Taichung 40201, Taiwan; 20 College of Medicine, National Taiwan University, Taipei 10617, Taiwan; 21 College of Life Science, National Taiwan University, Taipei 10617, Taiwan; 22 Ph.D. Program in Microbial Genomics, National Chung Hsing University, Taichung 402, Taiwan

**Keywords:** mitochondria, lung adenocarcinoma, *EGFR*-activating mutations, somatic mutations, prognosis

## Abstract

Risk factors including genetic effects are still being investigated in lung adenocarcinoma (LUAD). Mitochondria play an important role in controlling imperative cellular parameters, and anomalies in mitochondrial function might be crucial for cancer development. The mitochondrial genomic aberrations found in lung adenocarcinoma and their associations with cancer development and progression are not yet clearly characterized. Here, we identified a spectrum of mitochondrial genome mutations in early-stage lung adenocarcinoma and explored their association with prognosis and clinical outcomes. Next-generation sequencing was used to reveal the mitochondrial genomes of tumor and conditionally normal adjacent tissues from 61 Stage 1 LUADs. Mitochondrial somatic mutations and clinical outcomes including relapse-free survival (RFS) were analyzed. Patients with somatic mutations in the D-loop region had longer RFS (adjusted hazard ratio, adjHR = 0.18, *p* = 0.027), whereas somatic mutations in mitochondrial Complex IV and Complex V genes were associated with shorter RFS (adjHR = 3.69, *p* = 0.012, and adjHR = 6.63, *p* = 0.002, respectively). The risk scores derived from mitochondrial somatic mutations were predictive of RFS (adjHR = 9.10, 95%CI: 2.93–28.32, *p* < 0.001). Our findings demonstrated the vulnerability of the mitochondrial genome to mutations and the potential prediction ability of somatic mutations. This research may contribute to improving molecular guidance for patient treatment in precision medicine.

## 1. Introduction

Lung cancer is the leading cause of cancer death worldwide. Despite the advances in early detection and therapeutic methods, the 5 year survival rate for early-stage lung cancer remains about 55% [1]. Genomic aberrations associated with lung cancer development have been studied systematically in recent years [2]. Driver mutations in *EGFR*, *ALK*, and *ROS1* have been identified for non-smoking-associated LUAD [3]. *EGFR*-activating mutations are frequently observed in East Asian, female, and non-smoking LUAD patients [4,5]. For early-stage patients, surgery with or without adjuvant chemotherapy remains the therapeutic gold standard [6]. However, about 30–50% of early-stage patients develop disease relapse within 5 years of surgery, and identification of prognostic markers for risk of relapse remains a challenge [6]. 

The maternally inherited mitochondrion controls many imperative cellular functions, including regulation of energy production by facilitating oxidative phosphorylation system (OXPHOS), reactive oxygen species (ROS) regulation, adjusting cytosolic Ca ^+ +^ levels, modulation of oxidation and reduction status, and initiation of apoptosis [7]. In mammalian cells, mitochondria produce most of the ATP by facilitating OXPHOS. The human mitochondrial genome encodes for 13 key protein components of four OXPHOS complexes, while the other proteins required for mitochondrial function are encoded by the nuclear genome and imported into the mitochondria after post-translational modifications [8]. Unlike the nuclear genome, the mitochondrial genome lacks repair mechanisms, histones, and intronic regions, which makes it more susceptible to replication errors and damage by ROS and other environmental factors, leading to higher mutation frequency [9]. Regardless of the high mutation frequency, these mutations can be edifying for mitochondrial function. 

A haplogroup is a subgroup of a population who share a common ancestor. People in the same haplogroup share the same variants descendent from a common ancestor, and possess both geographical and ethnic-specific differences in prevalence [10]. Determination of maternal haplogroups via mitochondrial variants is the most successful method due to the polyploidy of the mitochondrial genome and the paucity of recombination [11]. The association of haplogroups and the prevalence of some cancers has been studied, although no such study has been performed for LUAD [12,13,14]. 

Most oncogenomic studies to date have intensively explored nuclear genomic mutations and overlooked the mitochondrial genome. The role of mitochondria in cancer progression has been investigated in a few cancer types, and it has been suggested that the acquisition of mitochondrial mutations impairing pathways of energy generation might be elemental for cancer progression [15,16]. Mutations in mitochondria were found to promote metastasis by modulating ROS production [17,18,19,20]. Decreased ATP production and apoptotic rate, increased cell growth, and increased risk of certain cancers have been associated with mitochondrial mutations [12,17,21,22]. For lung cancer, previous investigations attempting to delineate the role of mitochondrial mutations have been performed with PCR or microarray with small sample sizes, heterogeneity of stage, or patients from different ethnicities, and the associations of mitochondrial mutations and clinical outcomes were not profound [23,24]. Hence, evaluation of the predictive power of mitochondrial variants for clinical outcomes has not been conclusive so far. 

In this study, next-generation sequencing (NGS) approach was used to reveal the mitochondrial genomes of 61 Stage I LUAD patients, and to demonstrate the landscape of mutations in mitochondrial genome. We examined the association of mitochondrial mutations with clinical subgroups and clinical outcomes in order to understand the mitochondrial genomic anomalies. 

## 2. Results 

### 2.1. Clinical and Demographic Characteristics of Study Cohort

To profile mitochondrial mutations, mitochondrial genomes of 61 paired cancer and conditionally normal adjacent tissues were obtained and sequenced. Clinical and demographic characteristics of patients in the study are summarized in Table 1. Most patients (39 patients, 63.93%) had tumors larger than 2 cm. Out of the 61 patients, 42 (68.85%) patients had never smoked and 19 (31.15%) were current or previous smokers. Relapse was observed in 21 (34.4%) patients over the study period. A total of 36 (59.02%) patients were carriers of *EGFR*-activating mutations, and in 25 patients (40.98%), no *EGFR*-activating mutations were identified. The median age for patients in the study was 67.8 years (standard deviation ±12.1). 

### 2.2. Mitochondrial Mutational Spectrum

**Figure 1:**
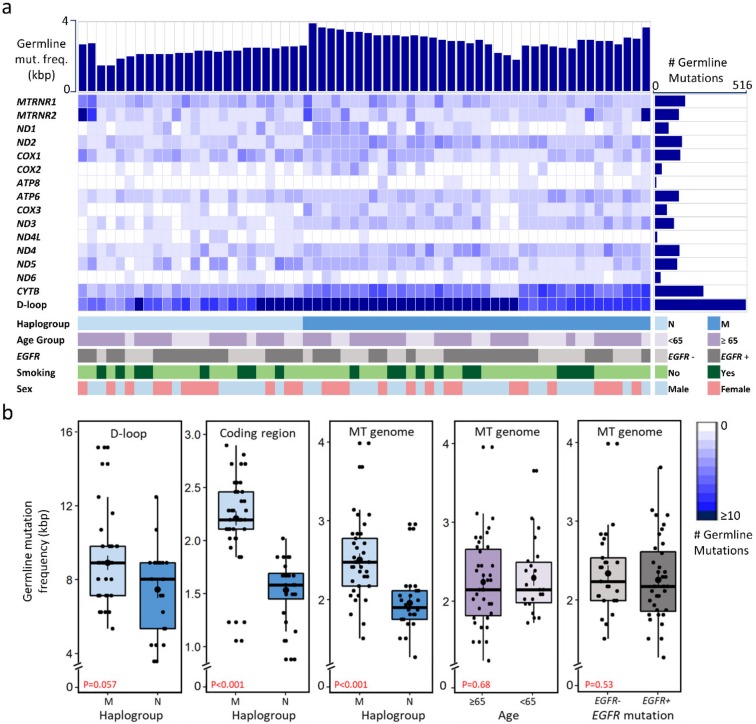
Identified germline mitochondrial mutations in early-stage lung adenocarcinoma patients. (**a**) Spectrum of germline mutations in 61 patients. Each row corresponds to a mitochondrial gene or region, and the color intensity indicates the number of mutations. (**b**) Germline mutation frequency (per kbp) in mitochondrial genomic regions with respect to haplogroup, age, and *EGFR*-activating mutation status. Abbreviations: *EGFR*+: patients harboring *EGFR*-activating mutations; *EGFR*−: patients without *EGFR*-activating mutations.

We identified 469 positions in the mitochondrial genome with 2180 germline mutations (Figure 1a, Figure S1, and Table S3). On average, each mitochondrial genome contained 36 germline mutations and the average mutation frequency was 2.29 mutations per kbp (range 1.26–3.98 per kbp, median 2.17 per kbp). The D-loop region was the most susceptible to germline mutations (23.81% mutations, mutation frequency of 8.34 per kbp), followed by the *CYTB* gene (20.65% mutations, mutation frequency of 3.97 per kbp). Among the protein-coding genes, *ND4L* and *ATP8* were the least mutated genes, with 15 and 11 germline mutations (mutation frequency of 0.83 and 0.95 per kbp), respectively. Most of the germline mutations in coding regions were synonymous (Table S4). 

The maternal haplogroups were identified from germline variants of the entire mitochondrial genome sequence. We classified patients in our study into haplogroup M (34 patients: lineages C, D, E, G, M7, M8, and M9) and haplogroup N (27 patients: lineages A, B, F, and N9; Table S5). 

We analyzed the distribution of germline mutations among the clinical variables and haplogroups (Figure 1b). It was apparent, as shown in Figure 1b, that haplogroup M accumulated more mutations in the entire D-loop region (*p* = 0.057), coding region (*p* < 0.001), and in the whole mitochondrial genome (*p* < 0.001). This pattern was consistent in mitochondrially encoded genes of Complex I (*p* < 0.001), Complex III (*p* < 0.001), and Complex IV (*p* = 0.012, Figure S2). In patients with *EGFR*-activating mutations, rRNA genes were highly mutated (*p* = 0.027), whereas *CYTB* or Complex III genes were highly mutated in patients without *EGFR*-activating mutations (*p* = 0.053, Figure S3). The differences in germline mutation frequencies between age, smoking status, and sex subgroups were not statistically significant (Figures S4, S5, and S6). Among other cancer types, the germline mutation frequencies were similar for mitochondrial protein-coding genes, and the D-loop was highly mutated, followed by Complex I genes (Figure S7). 

**Figure 2:**
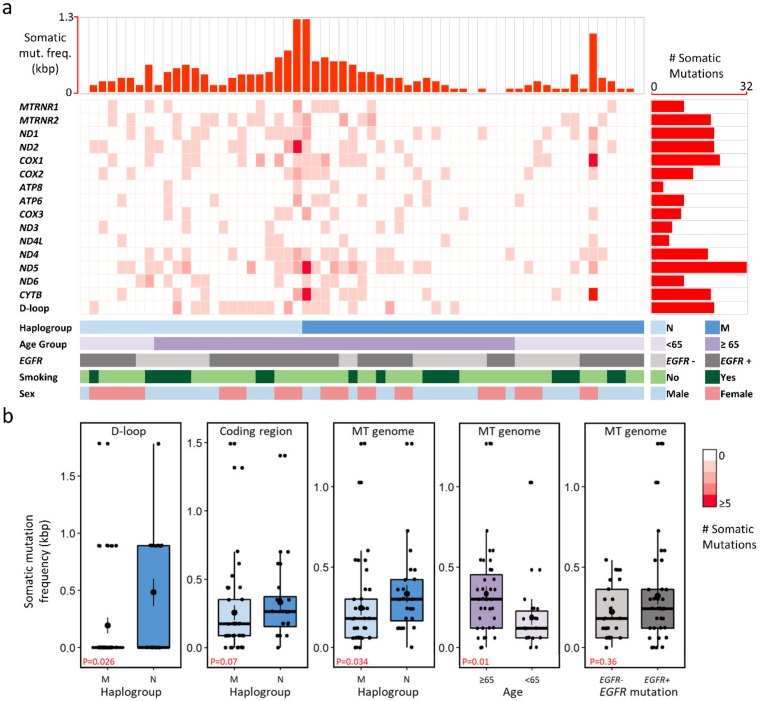
Somatic mutations identified in stage I LUAD patients. (**a**) Profile of mitochondrial somatic mutations; each column and row represent a sample and mitochondrial gene, respectively. The color intensity corresponds to the mutation count in a particular sample. (**b**) Somatic mutation frequency (per kbp) in mitochondrial genomic regions with respect to haplogroup, age, and *EGFR*-activating mutation status. Abbreviations- *EGFR*+: patients harboring *EGFR*-activating mutations, *EGFR*−: patients without *EGFR*-activating mutations.

To elucidate the variability of the mitochondrial genome in LUAD tumors, we analyzed their mutational spectra and identified 284 somatic mutations in the mitochondrial genomes of 56 (92%) patients (Figure 2a). The mean somatic mutation frequency was 0.28 mutation per kbp (range 0–1.2 per kbp, median 0.24 per kbp) and, on average, tumor genomes had four somatic mutations per patient. Most mutations (69.72%; 198/284) were observed in the coding regions, whereas D-loop region, tRNA, and rRNA genes accounted for fewer mutations collectively (30.28%; 86/284, Table S6). The *ND5* gene was found to be highly altered (32 genomic positions), followed by *COX1* gene (23 genomic positions, Figure 2a). The highest mutation frequency (0.37 per kbp) was observed in the D-loop region, followed by the mitochondrially encoded OXPHOS Complex I genes (0.30 per kbp, Table S6). About 64% of the somatic variants of OXPHOS complex genes were missense, and Complex I genes had the highest number of mutations (117/198 mutations), whereas Complex V genes were the least mutated (14/198 mutations, Table S7). 

Somatic mutation distribution was analyzed in haplogroups; interestingly, somatic mutations in haplogroups were observed to have contradictory pattern to that of germline mutations. A higher somatic mutation frequency was observed for haplogroup N than haplogroup M (*p* = 0.026, Figure 2b and Figure S8). The D-loop region was also identified to have significantly higher somatic mutation frequency in haplogroup N (*p* = 0.034). The frequency of somatic mutations in the *ND4* gene was higher in patients with *EGFR*-activating mutations (*p* = 0.053, Figure S9). 

We also confirmed the paradigm of increasing somatic mutation burden with patient age. As expected, the occurrence of somatic mutations was associated with patient age (*p* = 0.01). Patients over 65 years of age had a higher mutation frequency than patients younger than 65 (0.33 and 0.184 per kbp, respectively; *p* = 0.01, Figure 2b). The differences in mutation frequency of the two groups were evident in the coding regions as well as the D-loop region (Figure S10). Mutation frequency was not significantly different for smoking and sex subgroups (Figure S11 and S12). Profiles of somatic mutation frequency among mitochondrial protein-coding genes were similar for most cancer types (Figure S13). Among various cancer types, the highest mutation frequency was observed in lung squamous cell carcinoma followed by LUAD in the D-loop region and Complex I genes (Figure S13).

### 2.3. Nucleotide Substitution Profiles of Mitochondrial Mutations 

**Figure 3:**
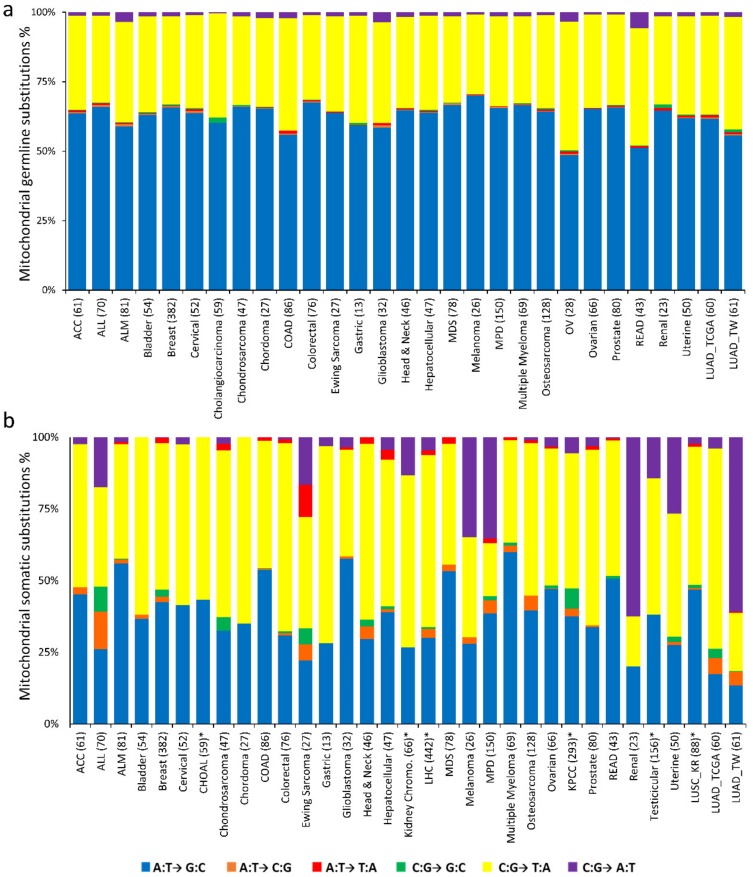
Nucleotide substitution profiles of mitochondrial mutations. (**a**) Nucleotide substitution profiles of germline mitochondrial mutations among different cancers. (**b**) Nucleotide substitution profiles of somatic mitochondrial mutations among different cancers. Cancer types marked with * provided the somatic mutation in the coding region only. Abbreviations: ACC: adenoid cystic carcinoma; ALL: acute lymphocytic leukemia; ALM: acute myeloid leukemia; CHOAL: cholangiocarcinoma; COAD: colon adenocarcinoma; Kidney Chromo.: kidney chromophobe; LHC: liver hepatocellular carcinoma; MDS: myelodysplastic syndromes; MPD: myeloproliferative disorders; OV: ovarian serous cystadenocarcinoma; KPCC: kidney renal papillary cell carcinoma; READ: rectum adenocarcinoma; LUAD_TCGA: lung adenocarcinoma TCGA; LUSC_KR: lung squamous cell carcinoma Korea; LUAD_TW: lung adenocarcinoma Taiwan.

We characterized germline mitochondrial nucleotide substitution profiles in this study and 23 other cancer datasets. The major proportion of the germline substitutions was contributed by A:T→G:C transitions, followed by C:G→T:A transitions. Most cancers had negligible proportions of C:G→G:C germline transversions (Figure 3a). 

The systematic evaluation of somatic mitochondrial nucleotide substitution profiles from this study and 34 other cancer datasets unveiled different nucleotide substitution patterns than in the germline profiles. Among all cancers, C:G→T:A transitions were the predominant substitution, whereas LUAD exhibited more C:G→A:T transversions (Figure 3b). The next most predominant substitution among most cancers was A:T→G:C transition followed by C:G→A:T transversions, whereas Taiwan LUAD had a prevalence of C:G→A:T followed by A:T→G:C. 

**Figure 4:**
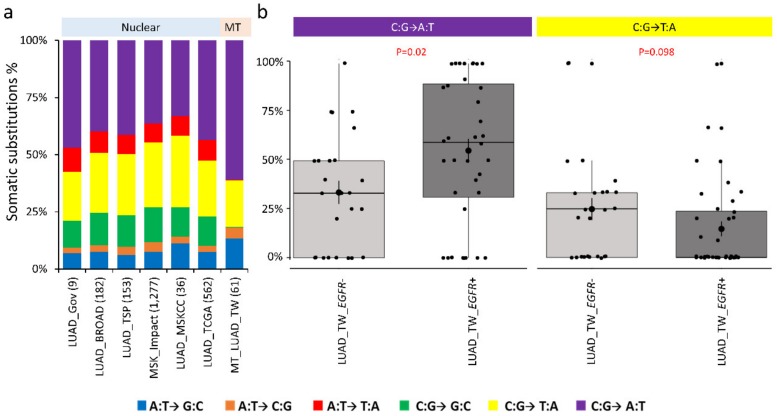
Comparison of nucleotide substitutions. (**a**) Comparison of somatic nucleotide substitution profiles of nuclear genomes from various studies and mitochondrial genomes. (**b**) Distribution of C to T transitions (G to A) and C to A (G to T) transversions among patients with and without *EGFR*-activation mutations.

Interestingly, distinct profiles of somatic mutations in mitochondrial and nuclear genome substitutions were observed in LUADs (Figure 4a). In both nuclear and mitochondrial genomes, the C:G→A:T transversions contributed most of the nuclear somatic mutations, followed by C:G→T:A transitions (Figure 4a). However, in the mitochondrial genome, the percentage of C:G→A:T was higher and C:G→T:A was lower than in the nuclear genome (both *p* < 0.001, Figure S14). The C:G→G:C transversions were nearly absent in mitochondrial genome, but accounted for about 12% of the nuclear somatic substitutions. 

In comparisons of clinical subgroups, nucleotide substitution profiles of mitochondrial somatic mutations among the smokers and non-smokers were significantly different for male and female (Figure S15). However, patients with *EGFR*-activating mutations had more C:G→A:T somatic substitutions in mitochondrial genomes than patients without *EGFR*-activating mutations (*p* = 0.02, Figure 4b). Additionally, in patients with *EGFR*-activating mutations, fewer C:G→T:A substitutions were observed (*p* = 0.098, Figure 4b). Further dissection of this association revealed that the C:G→T:A transitions were completely absent in the mitochondrial genomes of female patients without *EGFR*-activating mutations (Figure S16).

### 2.4. Mitochondrial Somatic Mutations and Prognosis of Lung Adenocarcinoma 

**Figure 5:**
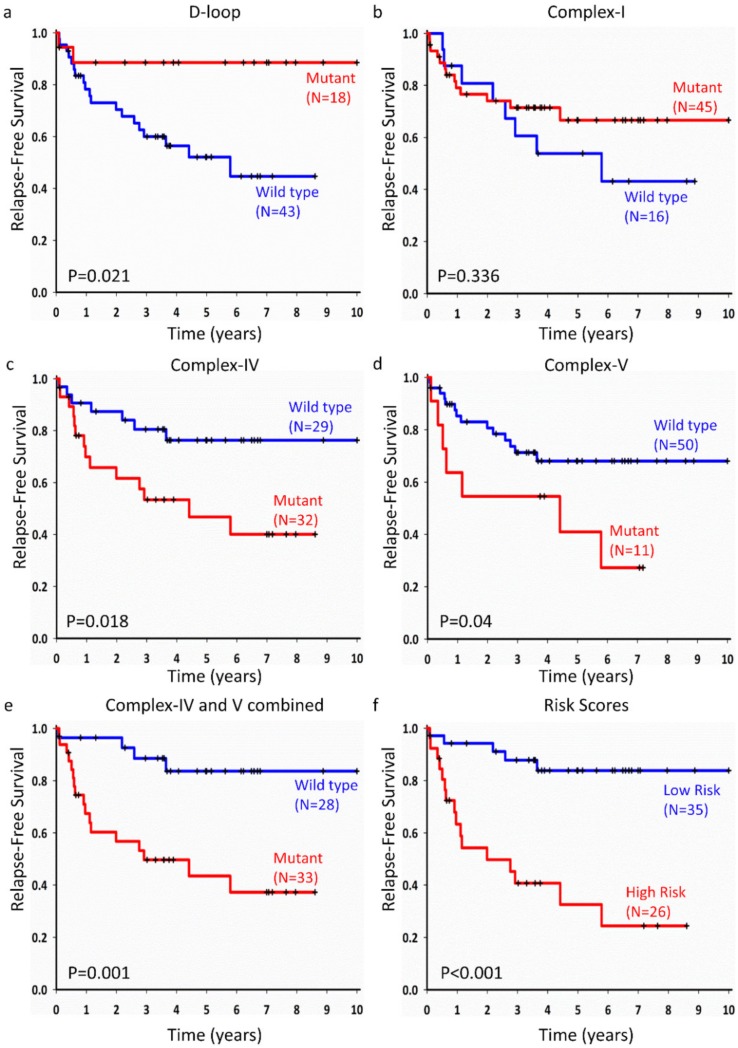
Kaplan–Meier estimation of relapse-free survival of early-stage lung adenocarcinoma patients. Mutations in the D-loop region (**a**) were associated with longer relapse-free survival, whereas mutations in Complex IV genes (**c**), Complex V genes (**d**), and Complex IV and Complex V combined (**e**) were associated with shorter relapse-free survival. No significant association was observed for Complex I mutations (**b**). For the risk-score-based stratification, patients with low risk scores showed better relapse-free survival (**f**). *p*-values were computed using the two-sided log-rank test.

We examined the associations of somatic mutations with prognosis of LUAD for individual genes, complexes, and the D-loop region. Patients with mutations in the D-loop region had longer RFS (median survival: not reached vs. 5.79 years, *p* = 0.021, Figure 5a), but showed no association with OS (Figure S17). For mitochondrially encoded Complex IV or Complex V genes, patients with mutations had shorter RFS (median survival: 4.41 years vs. not reached, *p* = 0.018, Figure 5c and median survival: 4.01 years vs. not reached, *p* = 0.018, Figure 5d, respectively), though no significant association with OS was identified (Figure S18, Table S7). For mutations in tRNA and rRNA regions, mitochondrially encoded Complex I, and Complex III genes, no significant associations were observed with either OS or RFS (Figure 5b, Figures S18 and S19). We estimated the combined effect of mutations in Complex IV and Complex V, and the results showed that mutations in the genes of these complexes demonstrated significant association with RFS (median survival: 2.92 years vs. not reached, *p* = 0.001, Figure 5e). 

The combined effects of mitochondrial variants in the D-loop region and OXPHOS complex genes were estimated by risk score method. Based on the median risk score, 35 patients were stratified into the low-risk group and 26 into the high-risk group. The low-risk group had longer RFS (median survival: 1.99 years vs. not reached, *p* = 0.001, Figure 5f), but no significant association was found with OS (Figure S18, Table S8).

To evaluate the independent prognostic value of the mitochondrial somatic mutations, Cox proportional hazards regression with covariate age, sex, tumor size, smoking, and *EGFR*-activating mutation status was performed. Results (Table 2) showed that mutations in the D-loop region, Complex IV, and Complex V were independently associated with RFS. Based on the adjusted hazard ratio, the mutations in D-loop region were protective (adjHR = 0.18, 95% CI: 0.04–0.82, *p* = 0.027), whereas mutations in Complex IV or Complex-V genes were risk-conferring (adjHR = 3.69, 95% CI: 1.34–10.18, *p* = 0.012 and adjHR = 6.63, 95% CI: 2.06–21.33, *p* = 0.001, respectively, Table 2). The combined effect of mutations in Complex IV and Complex V was also indicative of risk (adjHR = 8.63, 95% CI: 2.52–29.57, *p* = 0.001, Table 2). Patients stratified into the high-risk group had an adjusted hazard ratio of 9.01 (95% CI: 2.93–28.32, *p* < 0.001, Table 2). 

## 3. Discussion

Mitochondria are the primary site of energy production, and this energy generation is regulated by the interplay between mitochondrial and nuclear genomes [25]. Mitochondrial mutations and/or dysfunction play a crucial role in shifting cellular metabolism to a state more favorable for cancer proliferation [26,27]. This characteristic metabolic shift of tumor cells and increased glucose uptake has been previously described in breast cancer cells and pancreatic cancer [17,28]. Given the critical role of mitochondria in metabolism, somatic mutations in the mitochondrial genome might be important drivers of deregulated tumor metabolism. Despite the imperative function of mitochondria and the edifying effect of mitochondrial mutations, the mutational landscape of the mitochondrial genome has been relatively unexplored. Here, we reported mitochondrial mutation spectra of stage 1 LUAD patients, and the associations of these spectra with prognosis. 

In LUAD, for both germline and somatic mutations, the D-loop region was the most mutated non-coding region. *CYTB* and *ND* genes had highest mutation frequency among the protein-coding genes for germline and somatic mutations, respectively. The germline mutation frequency in OXPHOS complex genes for LUAD was similar to other cancer types; however, the somatic mutation frequency was different. These differences might have been caused by the sensitivity of methods used for sequencing and/or somatic mutation identification. Most studies to date have used aligned mitochondria reads from WES or WGS sequencing, and this disparity might be over/underrepresenting mitochondrial mutations (Figures S7, S13 and Table S1). 

Analysis of mutation distribution among the clinical subgroups identified intricate subgroup specific patterns for age, haplogroup, and *EGFR*-activating mutation status. The nexus of age and acquired mitochondrial mutations has been described in previous studies [7,9]. We also observed increased somatic mutation burden in patients of higher age. High Pol-G errors and lack of repair mechanism in mitochondria explain this mutation accretion with increasing age [9]. In the literature, scant data are available on mitochondrial haplogroups and incidence of cancer. Studies identifying association of *BRCA1* mutation with haplogroup M in China [12] and haplogroup X in Europe [21] have suggested a role of these haplogroups to present population-specific alterations in genes conferring risk to familial breast cancer. The identified lineages of M and N haplogroups in our patients are common in Asian and Chinese populations [29]. In our study, we observed haplogroup M to be more vulnerable to inherited mutation, whereas haplogroup N was more susceptible to acquired mutations. The distinguishing susceptibility of mitochondrial haplogroup for somatic and germline mutations requires further investigation. 

Mutations in the *EGFR* gene have been discovered to be strongly associated with lung cancers, especially LUAD [30]. LUAD patients with mutated *EGFR* have a significant response to TKI inhibitors [5]. Furthermore, in lung cancer cells, localization of *EGFR* onto mitochondria and its interaction with subunits of OXPHOS Complex IV have been characterized, although their function remains unknown [31,32]. The distribution of germline and somatic mutations was significantly different for particular genes with respect to the *EGFR*-activating mutation status. Higher germline mutation frequency was evident in the *CYTB* gene for patients without *EGFR*-activating mutations. On the other hand, *MTRNR2* (germline) and *ND4* (somatic) genes had higher mutation frequencies in patients with *EGFR*-activating mutations. The different association patterns of variants in protein-coding genes and rRNA genes with *EGFR* mutations indicate a distinct effect of *EGFR* mutations over the integrity of mitochondrial genome, and may have some functional implications as well.

The nucleotide substitution profiles unveiled different patterns of substitution in germline and somatic variants. Among all cancer types, transitions of A to G (or T to C) followed by C to T (G to A) were the predominant germline substitutions. A to G substitutions are frequently observed in mitochondrial genomes and might be due to the lack of proofreading activity in Pol-G [33]. In LUAD, C to A (G to T) transversions were the most common mitochondrial somatic substitutions. These changes are suggested to be induced by ROS damage through guanine oxidation [34]. The mutation frequency in LUAD_TCGA was low; few somatic mitochondrial mutations were identified in LUAD_TCGA (Figure S13) and the somatic nucleotide substitution profiles were thus different than inour dataset (Figure 3b). 

Interestingly, apart from LUAD and renal cancer, other cancers had low fractions of these C to A somatic transversions. The mitochondrial nucleotide substitution profiles of lung squamous cell carcinoma also demonstrated a large proportion of C to T mutations that might have been induced by smoking or tobacco exposure [35,36,37]. In contrast to the nuclear somatic nucleotide substitutions, the mitochondrial genomes presented distinguishing profiles. The absence of C to G (G to C) and high rate of C to A (G to T) transversions in mitochondrial genome implied more mutations with functional consequences (low transition/transversion ratio) [36]. In the nuclear genome, a substantial proportion of the mutations were C to T transitions, different from the C to A transversions observed in mitochondrial genomes. These different patterns imply distinct mutational processes in nuclear and mitochondrial genomes. 

We identified different association patterns of mitochondrial mutations with clinical outcomes. Patients carrying mutations in the D-loop were observed to have longer RFS, whereas shorter RFS was noted for carriers of mutations in mitochondrial OXPHOS genes (Figure 5). The estimated hazard ratios indicated that the mutations in the D-loop region were protective, whereas mutations in Complex IV and Complex V genes were not, with inimical effect for RFS. The mitochondrial D-loop is a main control region for coordinated replication and transcription of the mitochondrial genome, and accommodates two hypervariable regions that can form a peculiar-triple stranded structure [38]. In the present study, patients carrying mutations in the D-loop were observed to have longer RFS (Figure 5a, Table 2). The high average mutation frequency in D-loop region might have been due to the higher number of replication errors in the polymorphic repeat regions of the D-loop (Figure 1b, Table 1). These data, along with the high number of missense mutations in the coding region and control region, support the axiom of functional anomalies in mitochondria by somatic mutations. Although mutations in the D-loop region may impede mitochondrial replication and alter the transcription process, mechanisms of how these mutations influence cancer prognosis still need to be investigated. 

In our study, no association was observed between mitochondrial somatic mutation and OS (Table S8). All patients in our study were stage I and had undergone curative surgery for tumors. In the follow-up period of 10 years, patients with tumor recurrence also received drug or targeted therapies. The advancement in TKI-based therapies, radiotherapy, or chemotherapies for lung cancer patients could have been potential influential factors in the estimation of the association of OS with mitochondrial mutations. These patients were treatment-naïve for tumor relapse and, therefore, the analysis for association of RFS and mitochondrial variants did not have a confounding effect of treatment methods. 

We used our risk score assessment method on publicly available LUAD datasets. However, most datasets do not provide mitochondrial mutations. We obtained 37 samples from a LUAD_TCGA cohort with mitochondrial mutations and clinical data. The TCGA dataset lacked RFS data, meaning that we could not evaluate the performance of our method for RFS. No significant association was observed for OS in the TCGA_LUAD dataset, although the trend of OS in LUAD_TCGA was similar to that in our data (Figure S20)

Mutations in mitochondrial genes encoding for key subunits of OXPHOS complexes were associated with shortened RFS. Characterization of mutations in mitochondrially encoded OXPHOS complex genes provided evidence for their role in tumor development and progression. Previous studies have demonstrated that mitochondrial mutations are associated with elevated ROS production [27,39], increased invasion ability [40,41], decimated mitochondria copy number [39,42], and higher risk of breast cancer [21], oral squamous cell carcinoma [39,43], colorectal cancer [12,44], and thyroid oncocytoma [39,45]. The existence of empirical evidence for mitochondrial genomic mutations and subsequent mitochondrial dysfunction in several cancers suggests that mitochondrial mutations may have potential in evaluating the risk of cancer progression [19,20,28,39,41,43]. 

## 4. Materials and Methods

### 4.1. Study Population 

A total of 61 paired tumor and conditionally normal adjacent tissues of stage I LUAD patients were collected from Taichung Veteran General Hospital (Taichung, Taiwan) between April 2001 and April 2011. All patients received curative surgery with no adjuvant therapy. Median follow-up time was 4.2 years (50 months). Approval for this study was granted by the Institutional Review Board (Ethical Code：CF13083).

Mitochondrial somatic and germline variants from different cancer types were collected from cBioPortal, International Cancer Genome Consortium (ICGC), and published articles (Table S1). Clinical data of lung cancer samples were retrieved from Genomic Data Commons (GDC) data portal. Somatic nuclear genomic variants of LUAD were downloaded from GDC data portal (Table S2).

### 4.2. Mitochondrial Isolation, DNA Extraction, and Sequencing

Tumor and conditionally normal adjacent tissues were obtained from all participants in the study. For isolation of mitochondria, tissues (~200 mg) were ground with a mortar and pestle under liquid nitrogen. The fine powder was then transferred to homogenization medium (0.32 M sucrose, 1 mM EDTA, 10 mM Tris-HCl, pH 7.8, Sigma-Aldrich, USA) and centrifuged for 5 min at 1000 *g* until a homogenate mixture was obtained. To remove the nuclear material, homogenate mixture was centrifuged at 1000 *g* for 10 minutes and supernatant was transferred to 1.7 mL microcentrifuge tubes. For removal of remaining nuclear material, suspension of the pellet with equal volume of homogenization medium and centrifugation at 14,000 *g* for 10 minutes was repeated twice.

For extraction of mitochondrial DNA, the mitochondrial pellet was suspended in 600 µL lysis buffer (10 mM Tris-HCl, pH 8.0, 150 mM NaCl, 20 mM EDTA, 1% SDS, and 0.2 mg/mL Proteinase K) and incubated at 55 °C for 60 min (or until the lysate was clear). Repeated suspension, centrifugation at 13000 *g* for 5–15 minutes, and isolation of supernatant was done with equal volumes of buffer-saturated phenol (Invitrogen, Catalog# 15513-039, USA), phenol, chloroform, and mixture of isopropanol with 30 µg glycogen (Sigma-Aldrich, USA). The resultant pellet was allowed to dry for ~1 h, or until there were no visible droplets. These droplets were resuspended in 50–100 µL of 10 mM Tris, pH 8.0. All the centrifugation procedures were done at 4 °C. 

Isolated mitochondrial DNA pellets were then amplified by PCR. PCR-amplified DNA was ligated with an appropriate adapter and further subjected to library preparation. These libraries were then sequenced on an Illumina HiSeq 2000 platform to obtain paired-end reads of 100 bases in read length.

### 4.3. EGFR Genotyping

The *EGFR* mutation detection was performed via matrix-assisted laser desorption ionization time-of-flight mass spectrometry (MALDI-TOF MS), following the user’s manual for the MassARRAY^®^ system (SEQUENOM^®^, CA, USA). Customized primers and probes were used for detection and analysis of the *EGFR* mutations. After amplifying the genomic region containing the *EGFR* gene using PCR primers, single-nucleotide extension was performed using detection probes followed by MALDI-TOF MS analysis. The mutation frequency was calculated as % = (mutant-type height)/(mutant-type height + wild-type height) × 100, while height was obtained using Type4 software. For each sample, two to four duplications were performed and the average mutation frequency was used for further analysis. To reduce false positives due to contamination, sample preparation processes, and DNA extraction, MALDI-TOF MS analyses were performed by independent operators in a different area of the laboratory.

### 4.4. Bioinformatics Analysis 

Pretreatments of the sequencing data was done following standard methods. Quality checking for the sequenced reads was performed using FastQC version 0.11.3 (https://www.bioinformatics.babraham.ac.uk/projects/fastqc/) [46]. Adapter trimming and filtering of low-quality reads was done using NGSQCToolkit version 2.3.3 (http://www.nipgr.res.in/ngsqctoolkit.html) [47]. The sequencing reads were trimmed from the left for 10 bases observed to have relatively low quality. High-quality reads were aligned with the revised Cambridge reference sequence for human mitochondria (rCRS: Accession Number NC_012920.1) [48]. As the mitochondrial genome is circular, we used an adaptive approach to resolve the low depth of mapped reads in the D-loop region (position 16024 to576). The mitochondrial genome was divided into two parts to serve as a reference, one for the D-loop region and other for the mitochondrial genome devoid of D-loop. Along with the flanking regions in these reference genomes, read mapping was performed by the sub-optimal alignment module of BWA version 0.7.15 [49]. Alignment files were converted to BAM format using SAMtools version 0.1.19 [50]. Picard-Tools’ MarkDuplicates module (version 1.97) (https://broadinstitute.github.io/picard/) was used to remove PCR and optical duplicates. Germline mutations were identified by Genome Analysis Toolkit (GATK)-Haplotype caller version 3.6 [51]. PhyMer was used to identify haplogroups from the sequence alignment files of conditionally normal adjacent tissues [52]. Somatic mutations were identified using the Bayesian classifier approach incorporated intoGATK-Mutect2 version 3.6 [51]. Annotations for identified variants were added using the online version of the Variant Effect Predictor (VEP), which also predicts the in silico effect of non-synonymous variants [53]. Information regarding OXPHOS pathway components was obtained from KEGG pathway hsa00190 [8]. 

### 4.5. Statistical Analysis 

The frequencies of mitochondrial mutations among sexes, *EGFR*-activating mutation status, smoking status, and mitochondrial haplogroup were summarized to report the group-specific proportions. Mutation frequency for individual genes/genomic regions was calculated as the number of mutations in the gene/genomic region divided by the length of gene/genomic region for each sample. Average mutation load for an individual gene/complex was the average of mutation percentage in a particular gene/complex among all samples.

$$\mathrm{Mutation}\;\mathrm{Frequency}=\frac{m_{i\;}}{l}$$

$$\mathrm{Average}\;\mathrm{Mutation}\;\mathrm{Frequency}=\frac{1}{N}\overset{N}{\underset{i=1}{\sum }}\frac{m_{i}}{l}$$

$$\mathrm{Mutation}\;\mathrm{Load}=\frac{m_{i}}{M_{i}}$$

where *N* is the total number of samples, *m* is the number of mutations identified in an individual gene/genomic region or complex, *M* is the total number of mutations identified in a sample, and *l* is the length of the gene/genomic region or complex. 

Fisher’s exact test or Wilcoxon–Mann–Whitney tests for categorical or continuous data were used to compare the differences between groups. The Kaplan–Meier method was used to estimate survival curves and the difference between curves was evaluated by log-rank test. Hazard ratio (HR) estimated from univariate Cox proportional hazard regression was used to determine the prognostic effect. Variants showing significant associations with OS or RFS were then analyzed using multivariate Cox proportional hazard regression with covariates age, sex, *EGFR* mutation, tumor size, and smoking status to identify the independent prognostic mutations. 

The risk score method is a linear combination of weighting coefficients multiplied by corresponding mutation status (0 for no mutation and 1 for mutated). Weighting coefficients were derived from the univariate Cox proportional hazard regression coefficient of the D-loop region and individual mitochondrial genes in Complex IV and Complex V. The median of risk score was used as a cutoff to separate patients into high- or low-risk groups. 

All analyses were performed in SAS (version 9.4) or R studio (version 1.0.136). All tests were two-tailed and *p*-values < 0.05 were considered significant. 

## 5. Conclusions

Mutations in the mitochondrial genome may lead to dysregulated OXPHOS; the key to attaining Warburg phenotype. Anomalies in D-loop and OXPHOS genes showed association with prognosis of lung adenocarcinoma and revealed an undiscovered regime. Future functional studies are required to clarify the relationship between mitochondrial mutations and the prognosis of lung adenocarcinoma. Inclusion of mitochondrial-mutation-derived prognostic biomarkers may improve the prediction of patient outcome and triage of patients with high risk.

## Figures and Tables

**Table 1:** Clinical characteristics of 61 lung adenocarcinoma patients with Stage I disease.

Variables		# Patients	%	Low Risk	High Risk	*p*-val
Age						1
	=>65	39	63.9	22	17	
	<65	22	36.1	13	9	
Sex						0.1171
	Male	34	55.7	23	11	
	Female	27	44.3	12	15	
Smoking						0.2763
	Smokers	19	31.2	13	6	
	Never Smoked	42	68.9	22	20	
Tumor Size						0.5914
	<=2 mm	22	36.1	14	8	
	>2 mm	39	63.9	21	18	
*EGFR* mutation status						0.6001
	*EGFR +*	36	59	22	14	
	*EGFR −*	25	41	13	12	
Relapse						0.0003
	Relapse	21	34.4	5	16	
	No Relapse	40	65.6	30	10	
Survival						0.5626
	Survival	45	73.8	27	18	
	Death	16	26.2	8	8	

**Table 2:** Multivariate Cox regression analysis for relapse-free survival.

Variable	adjHR	95% CI		*p*-Value
D-loop				
Age	0.75	0.27	2.15	0.598
Sex	1.01	0.97	1.05	0.672
*EGFR* mutation	0.99	0.35	2.79	0.985
Tumor Size	3.74	0.99	14.07	0.051
Smoking Status	1.23	0.40	3.82	0.716
D-loop mutations	0.18	0.04	0.82	0.027
Complex I				
Age	0.99	0.95	1.03	0.573
Sex	0.89	0.31	2.57	0.827
*EGFR* mutation	0.78	0.28	2.16	0.633
Tumor Size	4.23	1.10	16.19	0.035
Smoking Status	0.99	0.32	3.04	0.983
Complex-I mutations	0.97	0.37	2.56	0.958
Complex IV				
Age	0.65	0.21	2.09	0.474
Sex	0.98	0.95	1.02	0.418
*EGFR* mutation	0.89	0.30	2.61	0.826
Tumor Size	3.99	1.13	14.14	0.032
Smoking Status	1.49	0.46	4.75	0.505
Complex-IV mutations	3.69	1.34	10.18	0.012
Complex V				
Age	0.62	0.21	1.81	0.379
Sex	0.97	0.93	1.01	0.141
*EGFR* mutation	0.58	0.19	1.73	0.324
Tumor Size	5.89	1.59	21.88	0.008
Smoking Status	1.05	0.32	3.37	0.941
Complex-V mutations	6.63	2.06	21.33	0.002
Complex IV and Complex V				
Age	0.60	0.18	1.95	0.391
Sex	0.98	0.94	1.02	0.325
*EGFR* mutation	0.91	0.31	2.72	0.869
Tumor Size	4.31	1.22	15.21	0.023
Smoking Status	2.07	0.63	6.86	0.233
Complex IV and V mutations	8.63	2.52	29.57	0.001
Risk Score				
Age	0.98	0.94	1.02	0.337
Sex	0.65	0.21	1.99	0.447
*EGFR* mutation	1.17	0.37	3.68	0.786
Tumor Size	4.03	1.11	14.66	0.034
Smoking Status	2.27	0.66	7.78	0.194
High risk group	9.10	2.93	28.32	<0.001

adjHR: adjusted hazard ratio, CI: confidence interval.

## Supplementary Materials

The following are available online at https://www.mdpi.com/2072-6694/12/3/755/s1, Figure S1: Positional distribution of germline mitochondrial variant in lung adenocarcinoma. Mitochondrial genomic components are represented by different colors, Figure S2: Germline mutation frequency of patients from haplogroup M and N: y-axis represents mutation frequency (per kbp) of corresponding gene or region, Figure S3: Germline mutation frequency of patients with and without *EGFR*-activating mutations, Figure S4: Germline mutation frequency among patients of age above and below 65 years, Figure S5: Germline mutation frequency among patients of different smoking habits i.e. never smokers and smokers, Figure S6: Germline mutation frequency among male and female patients, Figure S7: Germline mutation frequency (per kbp) of mitochondrial gene among different cancers types and the sequencing methods used, Figure S8: Somatic mutation frequency of haplogroup M and N patients: y-axis is mutation frequency (per kbp) of corresponding gene or region, Figure S9: Somatic mutation frequency in patients with and without *EGFR*-activating mutations, Figure S10: Somatic mutation frequency among patients of age above and below 65 years, Figure S11: Somatic mutation frequency among patients of different smoking habits i.e. never smokers and smokers, Figure S12: Somatic mutation frequency among male and female patients, Figure S13: Somatic mutation frequency (per kbp) of mitochondrial gene among different cancers types and the different sequencing methods used, Figure S14: Nucleotide substitution profiles of mitochondrial and nuclear genome for C:G→A:T and C:G→T:A substitutions, Figure S15: Gender wise nucleotide substitution profiles and mutation distribution among smoker and nonsmoker patients, Figure S16: Gender wise nucleotide substitution profiles among patients with or without *EGFR* activating mutations, Figure S17: Kaplan-Meier estimation of overall survival of stage-I lung adenocarcinoma patients for mutations in individual mitochondrial genes and regions, Figure S18: Kaplan-Meier estimation of Relapse-free survival of early stage lung adenocarcinoma patients for mutations in individual mitochondrial genes, Figure S19: Kaplan-Meier estimation of overall survival of early stage lung adenocarcinoma patients for mutations in D-loop region and mitochondrial encoded OXPHOS complex genes, Figure S20: Kaplan-Meier estimation of overall survival for LUAD_TCGA cohort, Table S1: External cohorts used for mitochondrial nucleotide substitution profiles and mutation frequency estimation, Table S2: External cohorts used for comparison of nuclear genome somatic nucleotide substitution profile, Table S3: Summary of identified germline mutations in mitochondrial genome, Table S4: Distribution and nature of germline mutations in mitochondrial encoded OXPHOS complex genes, Table S5: Identified haplogroups and lineages in 61 lung adenocarcinoma patients, Table S6: Summary of identified somatic mutations in mitochondrial genome, Table S7: Distribution and nature of somatic mutations in mitochondrial encoded OXPHOS complex genes, Table S8: Multivariate Cox Regression analysis for overall survival.
