# Supplementary material for: Landscape of Mitochondria Genome and Clinical Outcomes in Stage 1 Lung Adenocarcinoma
Source: Cancers (Basel). 2020 Mar 23;12(3):755. doi: 10.3390/cancers12030755 (PMC7140061; doi:10.3390/cancers12030755)
Supplement: Supplementary file 1 [file cancers-12-00755-s001.pdf]

Supplementary Material: Landscape of Mitochondria Genome and Clinical Outcomes in Stage 1 Lung adenocarcinoma

Table S1: External cohorts used for mitochondrial nucleotide substitution profiles and mutation frequency estimation

Cancer Type	Sequencing	#Samples	References
Acute Lymphocytic Leukemia	WES+WGS	70	[33]
Acute Myeloid Leukemia	WES+WGS	81	[33,54]
Adenoid Cystic Carcinoma	WES+WGS	61	[33]
Bladder	WGS	54	[33]
Breast	WES+WGS	382	[33]
Cervical	WES	52	[33]
Cholangiocarcinoma*	WES	59	[33,42]
Chondrosarcoma	WES	47	[33]
Chordoma	WES+WGS	27	[33]
Colan adenocarcinoma	WES	86	[54]
Colorectal	WES+WGS	76	[33]
Ewing sarcoma	WES	27	[33]
Gastric	WES	13	[33]
Glioblastoma	WES	32	[54]
Head & Neck	WES+WGS	46	[33]
Hepatocellular	WES	47	[33]
Liver Hepatocellular Carcinoma*	WES	442	[55]
Kidney Chromophobe*	WES	66	[56]
Kidney Renal Papillary Cell Carcinoma*	WES	293	[57]
Liver Hepatocellular Carcinoma	WES	442	[33]
Lung (LUAD-TCGA)	WGS	60	[33]
Lung Squamous Cell Carcinoma (LUSC_KR)	WES	88	[58]
Melanoma	WES+WGS	26	[33]

Multiple myeloma	WES	69	[33]
Myelodysplastic Syndromes	WES+WGS	78	[33]
Myeloproliferative Disorders	WES+WGS	150	[33]
Osteosarcoma	WES+WGS	128	[33]
Ovarian	WES	38	[33]
Ovarian Serous Cystadenocarcinoma	WES	28	[54]
Prostate	WGS	80	[33]
Rectal adenocarcinoma	WES	43	[54]
Renal	WES	23	[33]
Testicular Germ Cell Cancer	WES	156	[55]
Uterine	WES+WGS	50	[33]

Studies marked with * provided coding region somatic variants only, thus were not included in germline analysis

Table S2: External cohorts used for comparison of nuclear genome somatic nucleotide substitution profile

	Remarks	#Samples	Ref.
Broad Institute	Lung adenocarcinoma dataset	182	[30]
MSKCC	Lung adenocarcinoma dataset	34	[59]
TCGA LUAD	Lung adenocarcinoma dataset	566	[60]
TSP LUAD	Lung adenocarcinoma dataset	153	[61]
MSK Impact*	Filtered lung adenocarcinoma samples	1,277	[62]
NSCLC (Govindan et al.)	Filtered lung adenocarcinoma samples	9	[63]

*MSK Impact is cancer panel study, with sequencing information of 300 genes

Table S3: Summary of identified germline mutations in mitochondrial genome

	#Mutations	%	AMF
Total mutations	2,180	-	2.29
D-loop	516	23.81	8.34
tRNA & rRNA genes	327	14.97	1.94
OXPHOS Complex genes	1,327	60.76	1.62
Complex-I genes	653	49.21	1.73
Complex-III genes	274	20.65	3.97
Complex-IV genes	253	19.07	1.4
Complex-V genes	147	11.08	2.73

*AMF is average mutation frequency (per kbp) for corresponding genomic region and is calculated as the total number of mutations divided by the length of mitochondrial genomic region.

Table S4: Distribution and nature of germline mutations in mitochondrial encoded OXPHOS complex genes

Type of mutations	Complex-I(%)	Complex-III(%)	Complex-IV(%)	Complex-V(%)
Missense	172 (27)	133 (49)	25 (10)	134 (91)
Synonymous	476 (73)	142 (42)	227 (90)	13 (9)
Others	2 (0)	0	1 (0)	0
Total # Mutations	653	274	253	148

Table S5: Identified haplogroups and lineages in 61 lung adenocarcinoma patients

Haplogroup	Lineage	# Patients
M	M7	11
	D	12
	C7	3
	M8	3
	G	2
	E1	1
	M9	2
N	B4	6
	N9	3
	B5	4
	A	2
	F	12

Table S6: Summary of identified somatic mutations in mitochondrial genome

	#Mutations	%	AMF
Total mutations	284	-	0.28
D-loop	21	7.39	0.31
tRNA & rRNA genes	65	22.89	0.27
OXPHOS Complex genes	198	69.72	0.29
Complex-I genes	117	59.09	0.3
Complex-III genes	20	10.1	0.29
Complex-IV genes	47	23.74	0.26
Complex-V genes	14	7.07	0.26

*AMF is average mutation frequency (per kbp) for corresponding genomic region and is calculated as the total number of mutations divided by the length of mitochondrial genomic region.

Table S7: Distribution and nature of somatic mutations in mitochondrial encoded OXPHOS complex genes

Type of mutations	Complex-I(%)	Complex-III(%)	Complex-IV(%)	Complex-V(%)
Missense	75 (64)	12 (60)	31 (66)	10 (71)
Synonymous	35 (30)	6 (30)	13 (28)	3 (21)
Others	7 (6)	2 (10)	3 (6)	1 (7)
Total # Mutations	117	20	47	14

Table S8: Multivariate Cox Regression analysis for overall survival

Variable	Ad. HR	95% CI		P-value
		D-loop		
Age	1.0248	0.9799	1.072	0.2842
Sex	0.2225	0.04086	1.211	0.0822
EGFR mutation	1.3638	0.38684	4.808	0.6293
Tumor Size	1.4951	0.43144	5.181	0.5259
Smoking Status	3.807	1.1614	12.479	0.0273
D-loop mutation	0.4362	0.12157	1.565	0.203
		Complex-I		
Age	1.0065	0.9626	1.052	0.7773
Sex	0.2376	0.04396	1.285	0.0951
EGFR mutation	1.3572	0.39329	4.683	0.6289
Tumor Size	1.5523	0.44294	5.44	0.4919
Smoking Status	3.9423	1.16378	13.355	0.0276
Complex-I mutations	1.5364	0.4386	5.382	0.502
		Complex-IV		
Age	1.0081	0.96481	1.053	0.7197
Sex	0.2283	0.04086	1.276	0.0924
EGFR mutation	1.3369	0.39281	4.55	0.6422
Tumor Size	1.4361	0.42501	4.853	0.5601
Smoking Status	3.9716	1.21627	12.969	0.0224
Complex-IV mutations	1.3586	0.46143	4	0.5781
		Complex-V		
Age	1.0029	0.96049	1.047	0.8965
Sex	0.1727	0.03007	0.992	0.049
EGFR mutation	1.4016	0.37714	5.209	0.6142
Tumor Size	1.722	0.49335	6.01	0.3941
Smoking Status	4.8776	1.29789	18.33	0.019

Complex-V mutations	4.0698	0.86687	19.107	0.0753
Complex-IV and Complex-V				
Age	1.0012	0.95667	1.048	0.9575
Sex	0.1992	0.03449	1.15	0.0713
EGFR mutation	1.3216	0.39634	4.407	0.65
Tumor Size	1.6616	0.49033	5.631	0.4148
Smoking Status	4.3126	1.31586	14.134	0.0158
Complex-IV and V mutations	2.283	0.75352	6.917	0.1444
Risk Score				
Age	1.0028	0.95917	1.048	0.9008
Sex	0.1992	0.03539	1.122	0.0673
EGFR mutation	1.3807	0.39578	4.817	0.6128
Tumor Size	1.5869	0.45284	5.561	0.4705
Smoking Status	4.0527	1.21822	13.482	0.0225
Risk Groups	2.3519	0.82248	6.725	0.1106

Ad. HR: Adjusted Hazard Ratio, CI: Confidence Interval

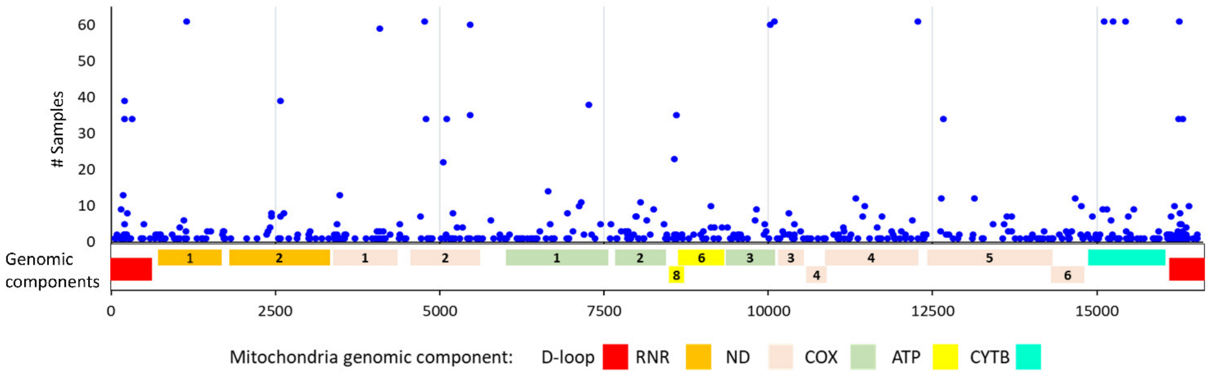

Figure S1: Positional distribution of germline mitochondrial variant in lung adenocarcinoma. Mitochondrial genomic components are represented by different colors.

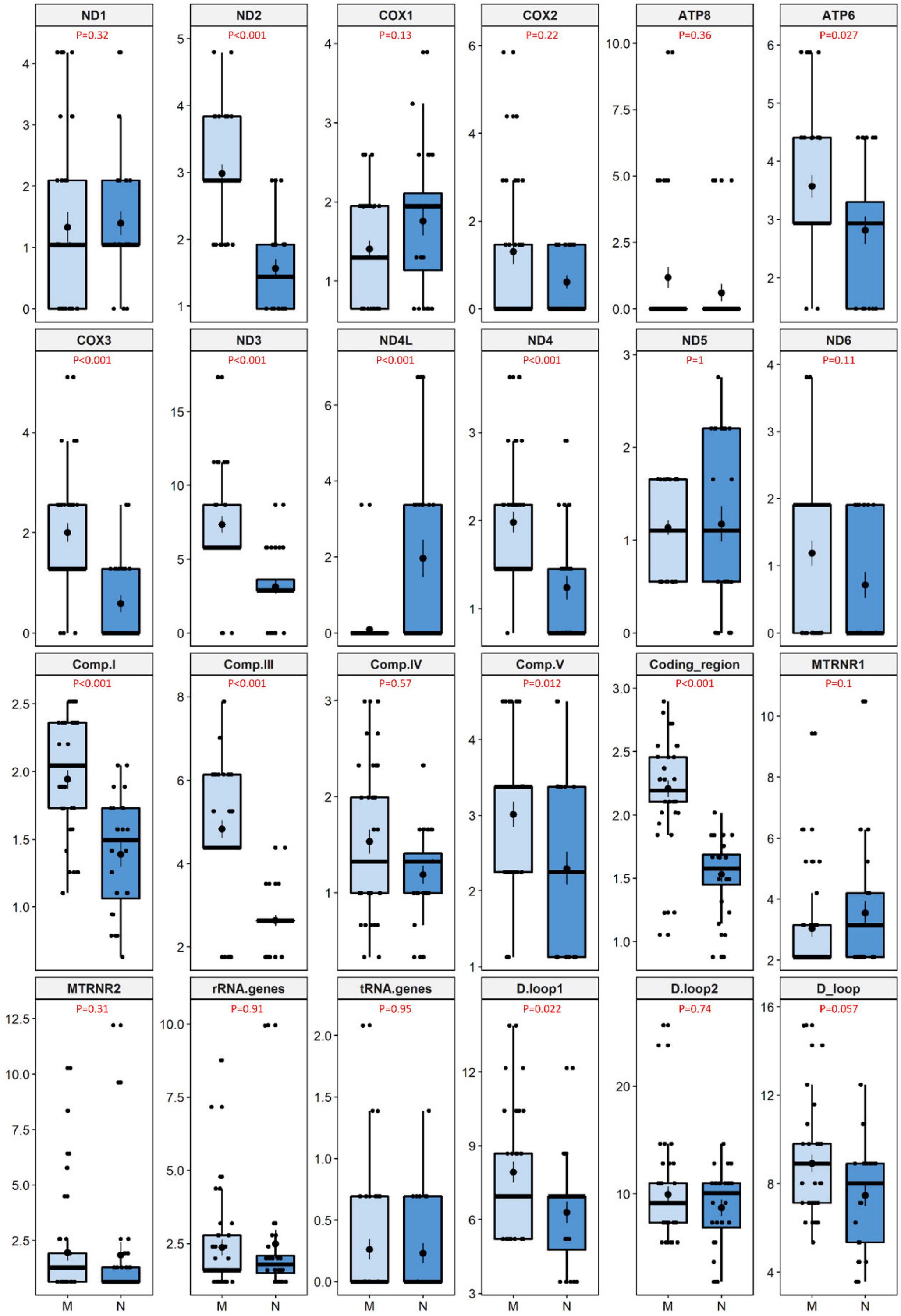

Figure S2: Germline mutation frequency of patients from haplogroup M and N: y-axis represents mutation frequency (per kbp) of corresponding gene or region.

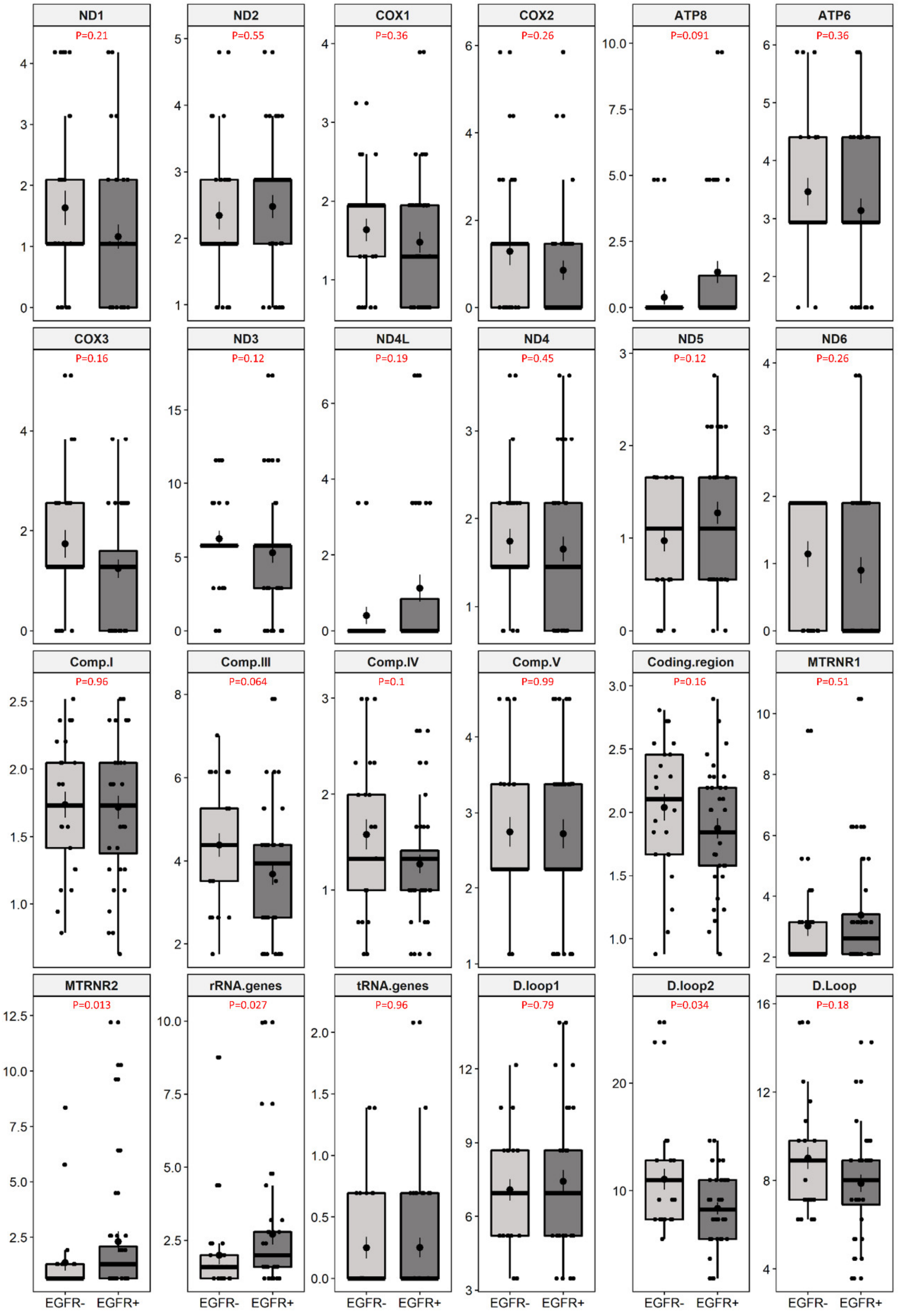

Figure S3: Germline mutation frequency of patients with and without *EGFR*-activating mutations (*EGFR*+ and *EGFR*- respectively): y-axis is mutation frequency (per kbp) of corresponding gene or region.

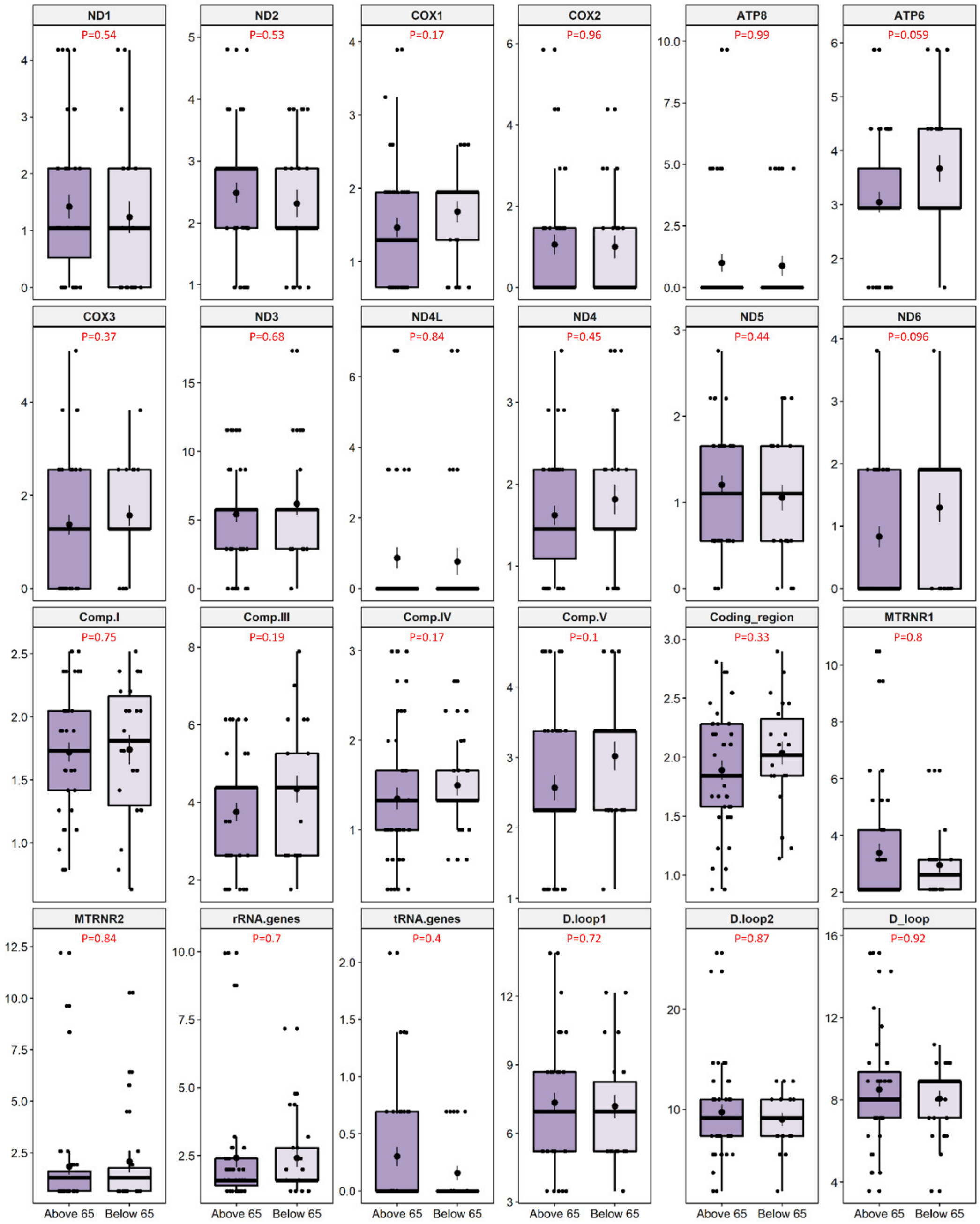

Figure S4: Germline mutation frequency among patients of age above and below 65 years. y-axis is mutation frequency (per kbp) of corresponding gene or region.

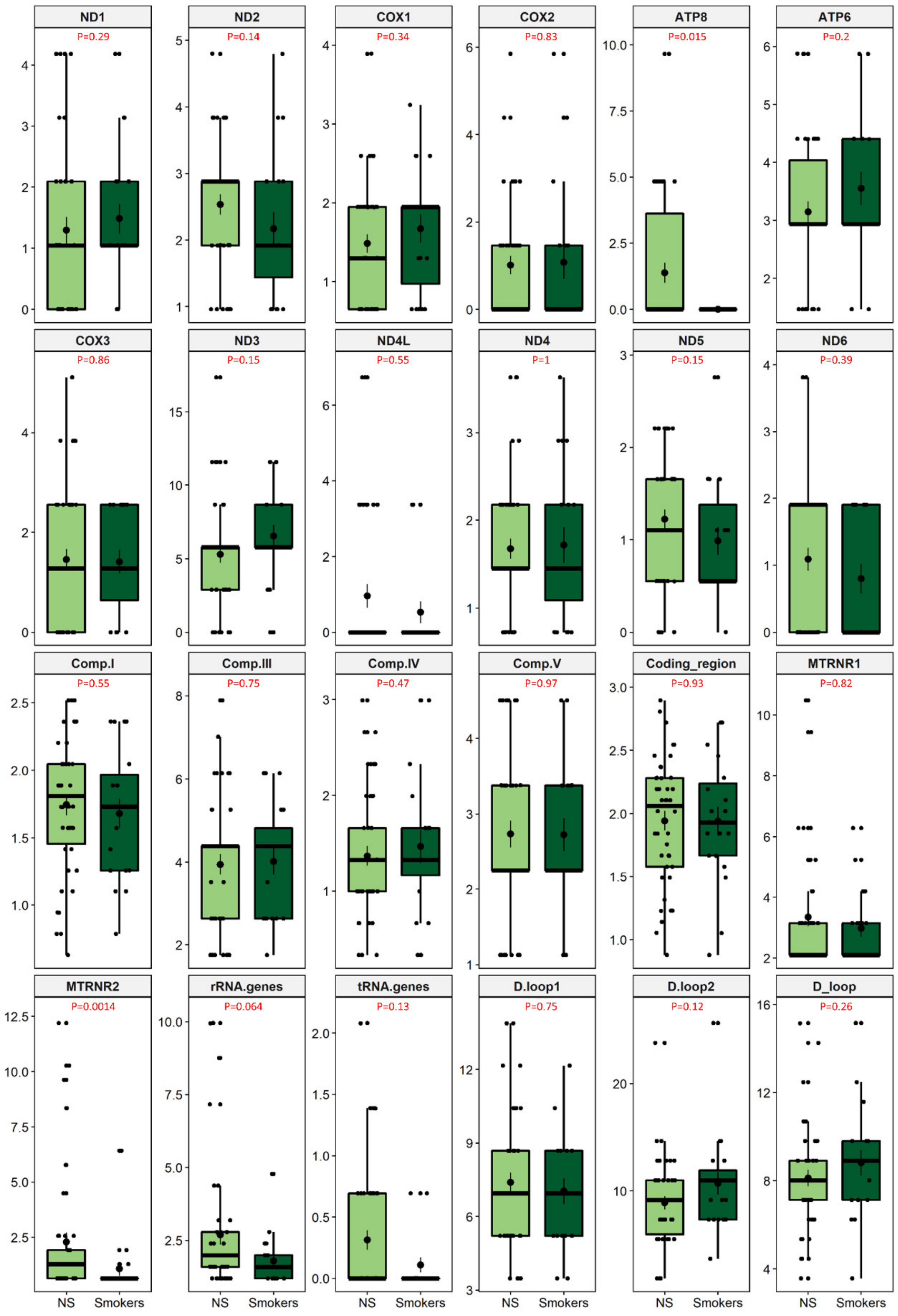

Figure S5: Germline mutation frequency among patients of different smoking habits i.e. never smokers and smokers (NS and Smokers, respectively): y-axis is mutation frequency (per kbp) for corresponding gene or region.

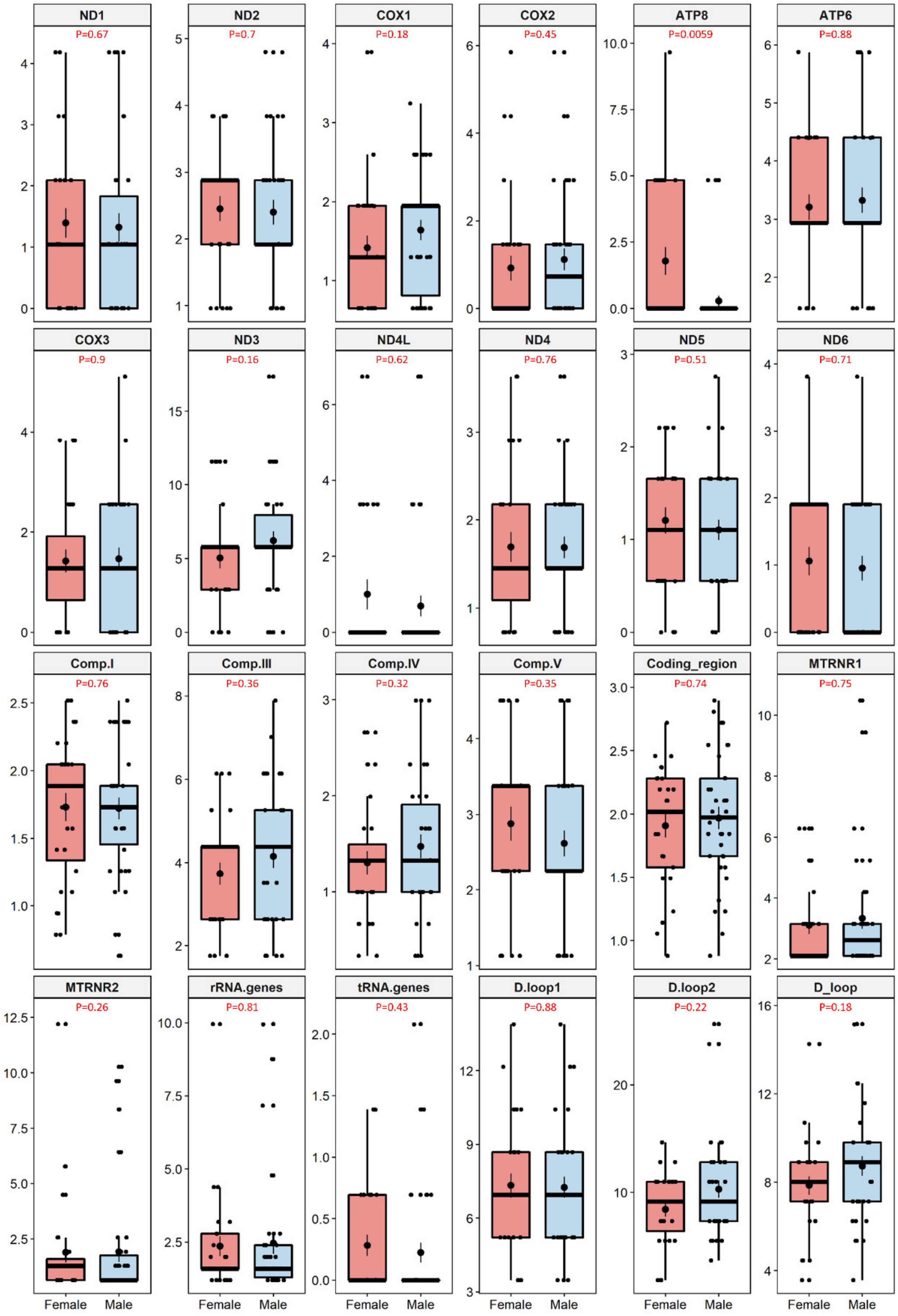

Figure S6: Germline mutation frequency among male and female patients: y-axis is mutation frequency (per kbp) of corresponding gene or region

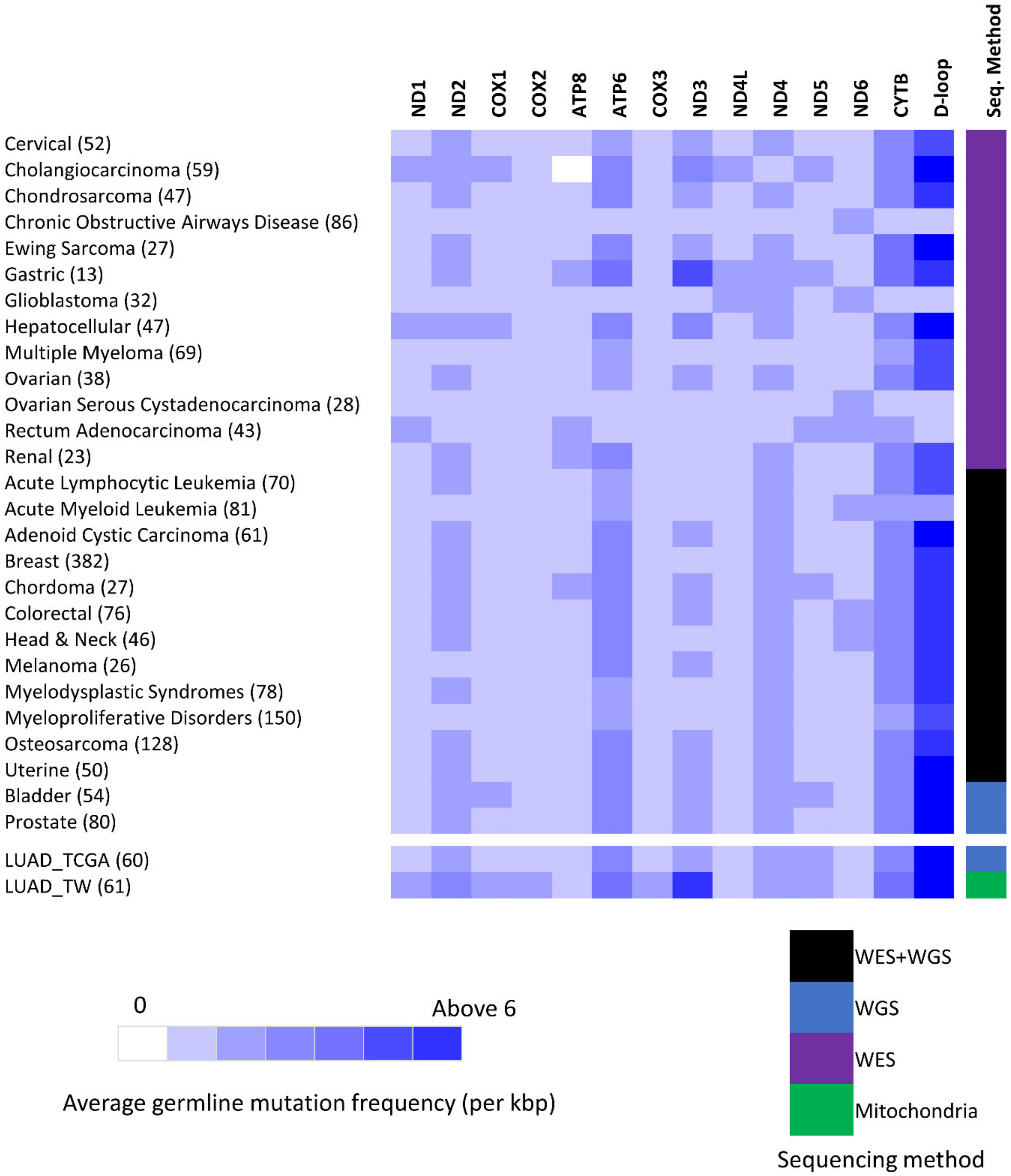

Figure S7: Germline mutation frequency (per kbp) of mitochondrial gene among different cancers types and the sequencing methods used.

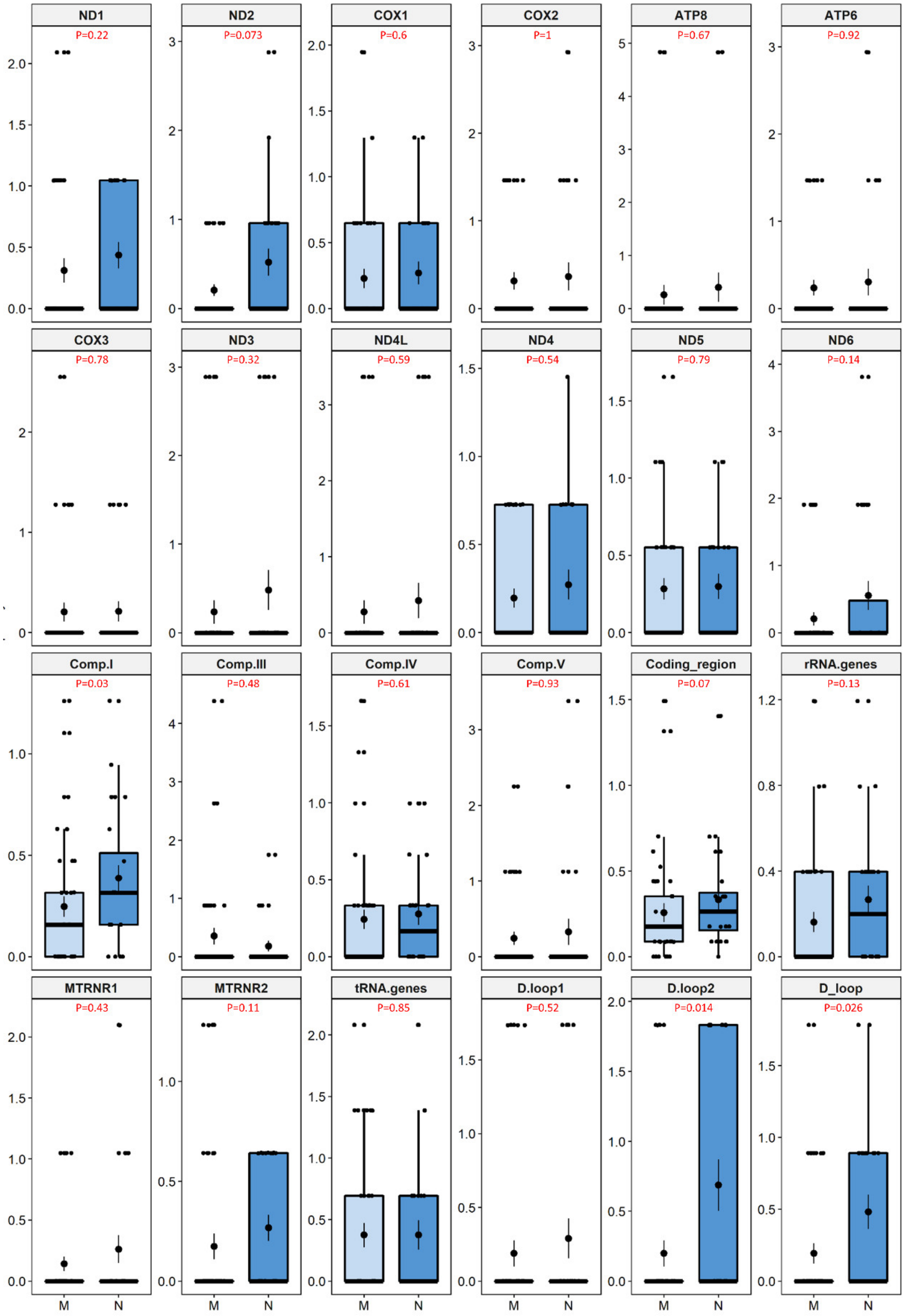

Figure S8: Somatic mutation frequency of haplogroup M and N patients: y-axis is mutation frequency (per kbp) of corresponding gene or region.

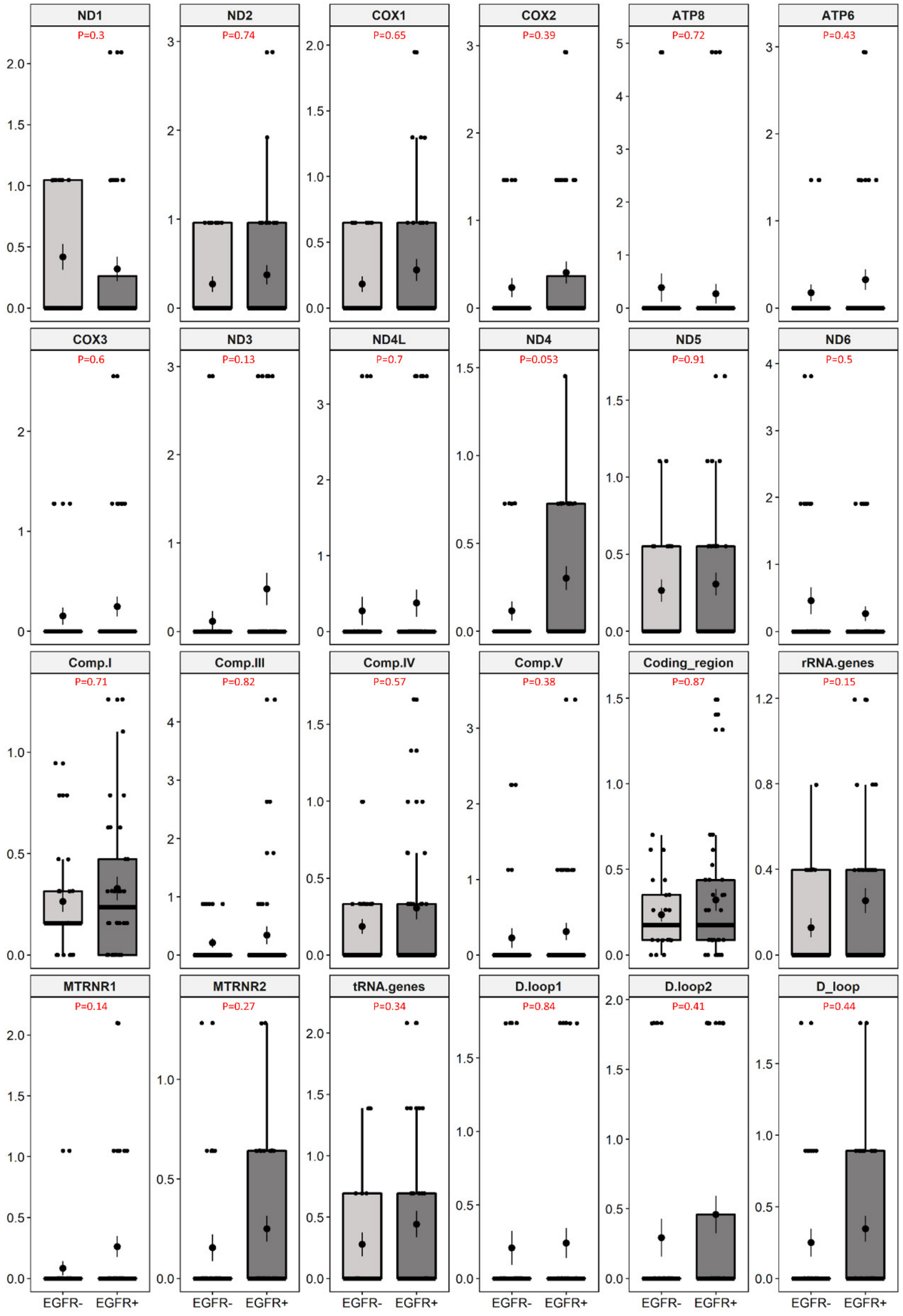

Figure S9: Somatic mutation frequency in patients with and without *EGFR*-activating mutations (*EGFR*+ and *EGFR*- respectively): y-axis represents mutation frequency (per kbp) of corresponding gene or region.

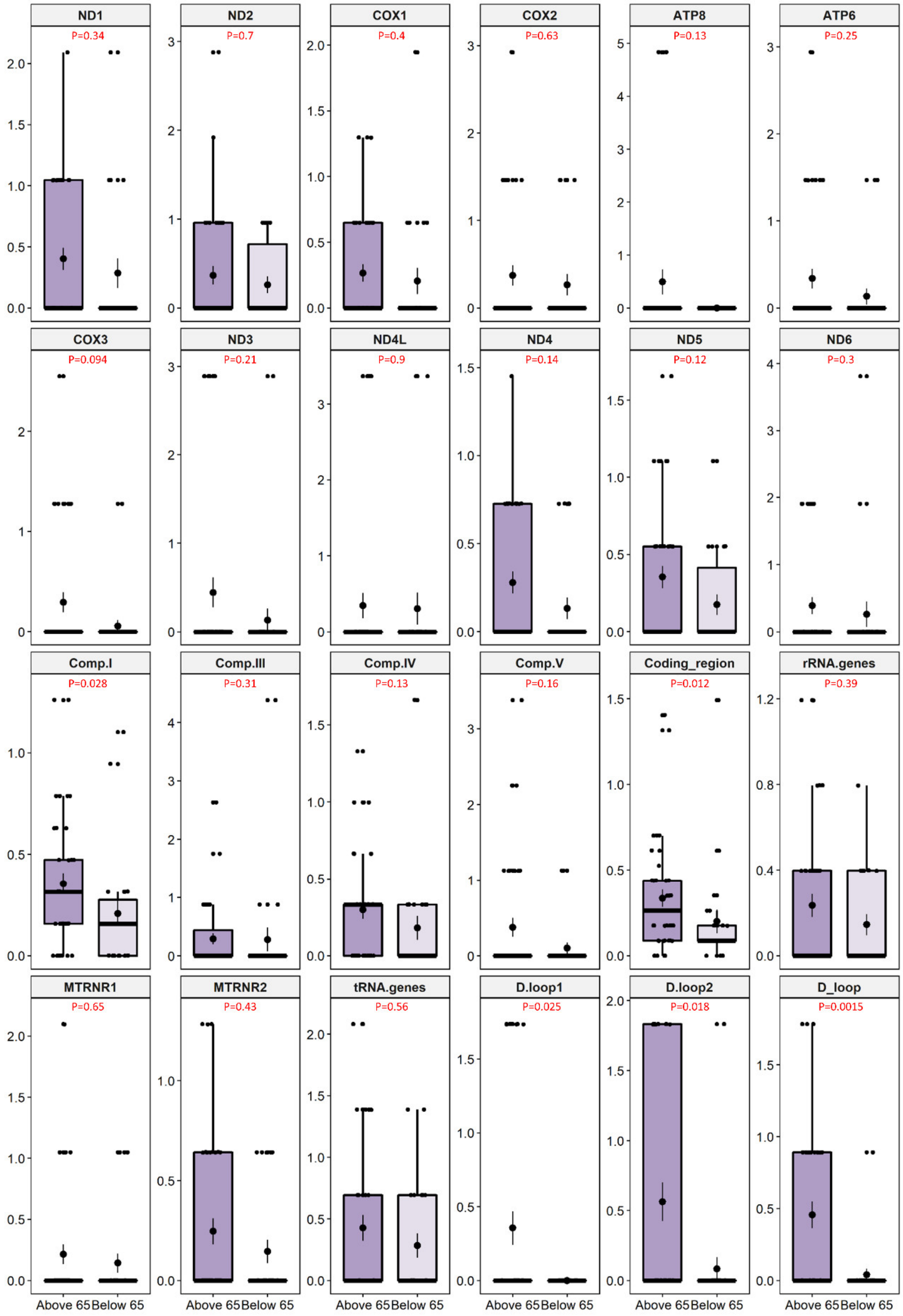

Figure S10: Somatic mutation frequency among patients of age above and below 65 years. y-axis is mutation frequency (per kbp) of corresponding gene or region.

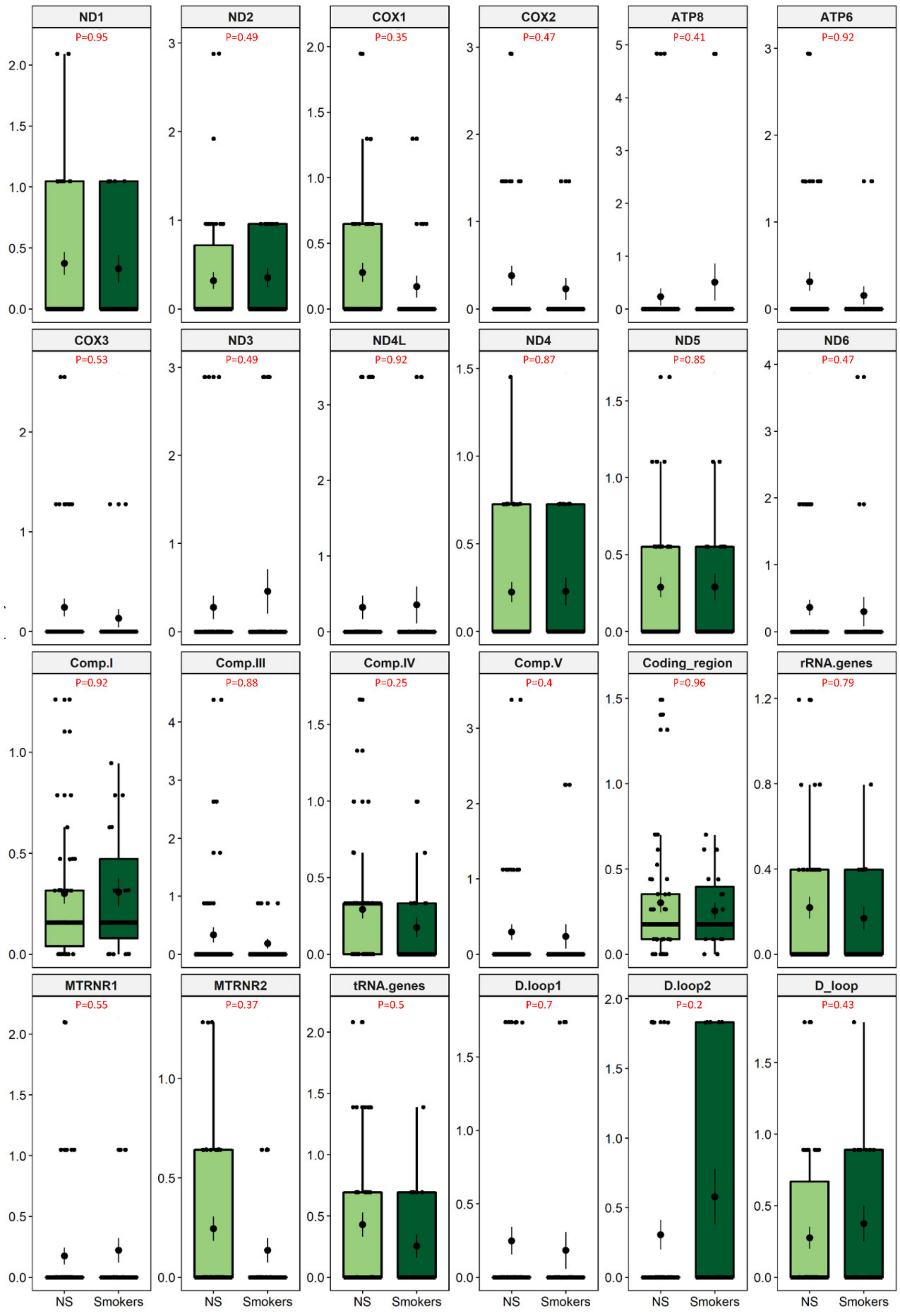

Figure S11: Somatic mutation frequency among patients of different smoking habits i.e. never smokers and smokers (NS and Smokers, respectively): y-axis is mutation frequency (per kbp) of corresponding gene or region.

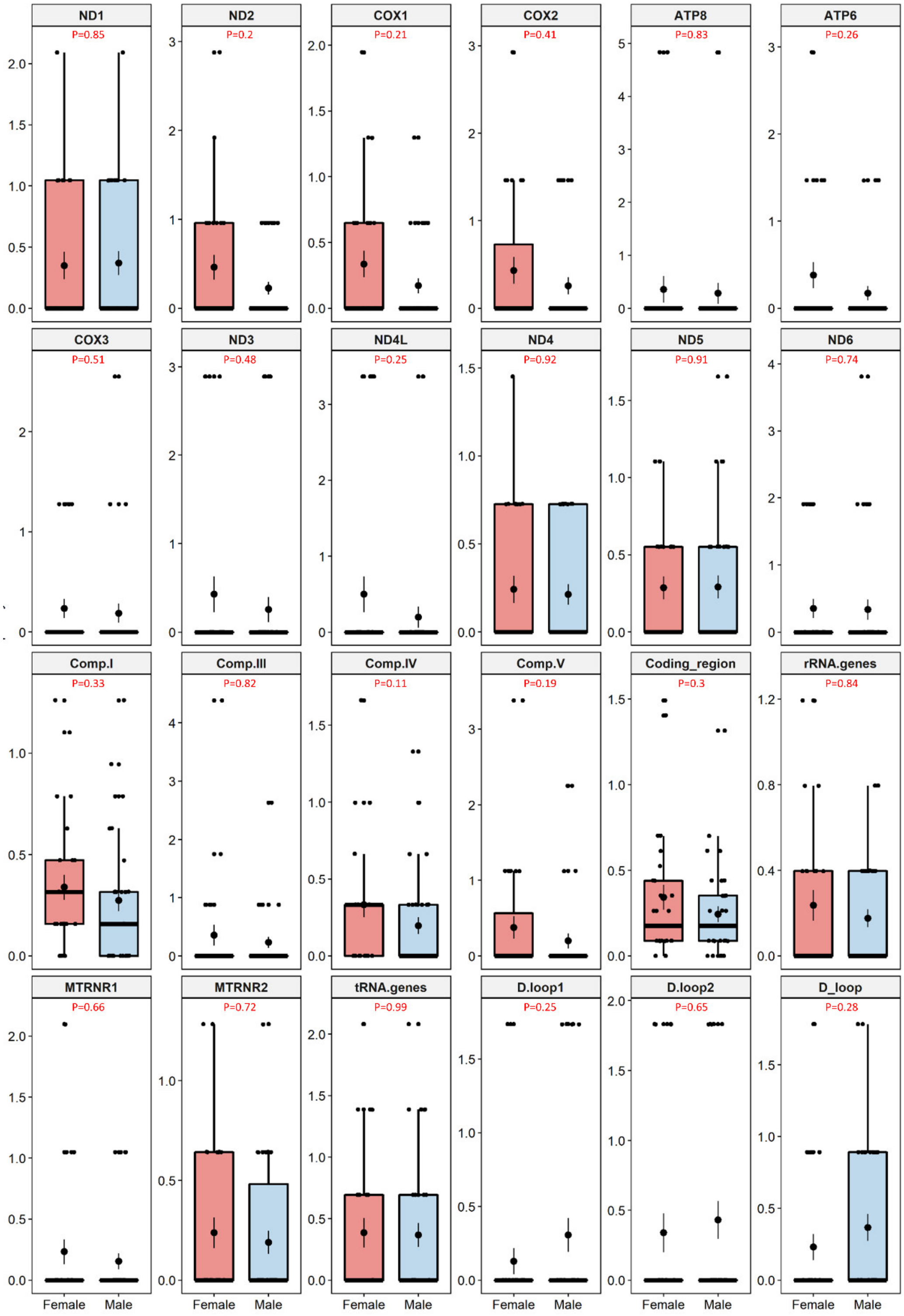

Figure S12: Somatic mutation frequency among male and female patients: y-axis is mutation frequency (per kbp) of corresponding gene or region.

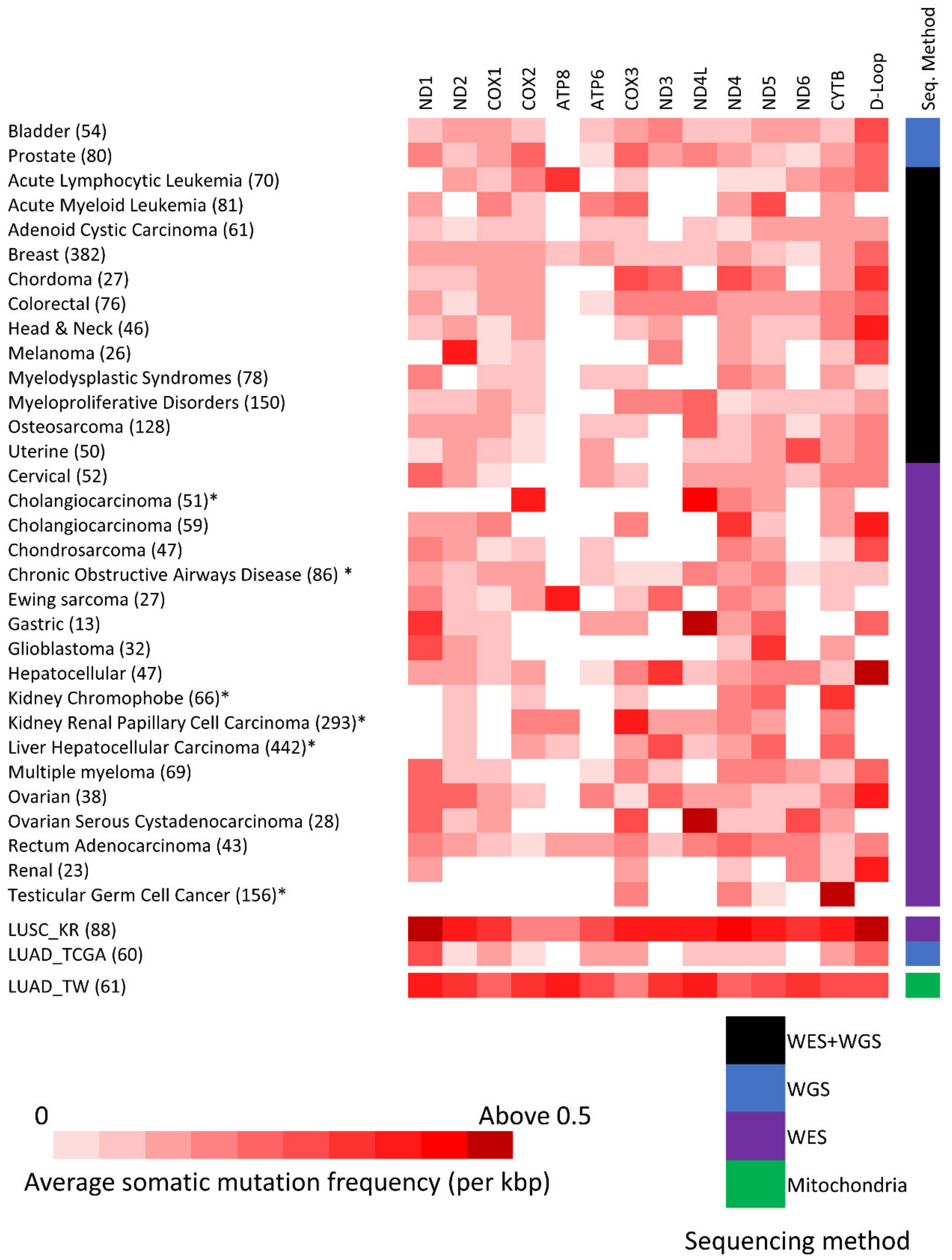

Figure S13: Somatic mutation frequency (per kbp) of mitochondrial gene among different cancers types and the different sequencing methods used.

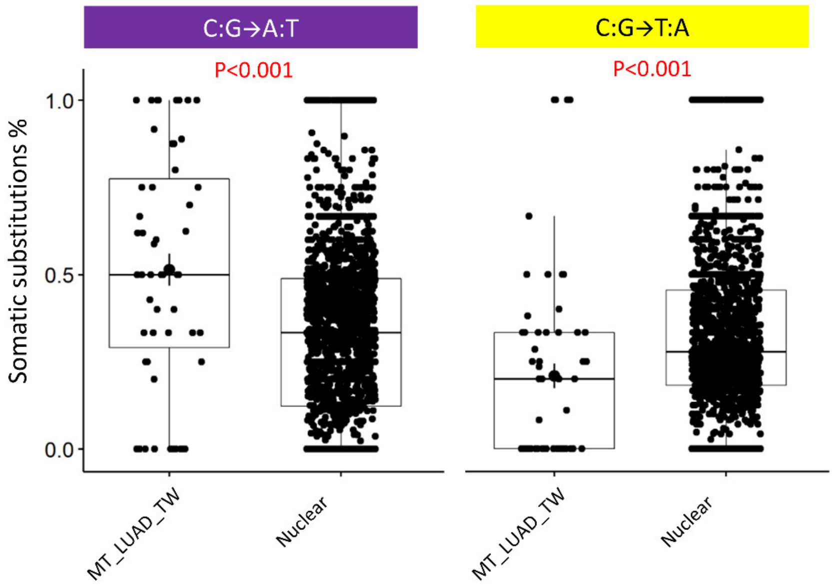

Figure S14: Nucleotide substitution profiles of mitochondrial and nuclear genome for C:G→A:T and C:G→T:A substitutions. The nuclear genome data is aggregate of datasets listed in Supplementary Table 2.

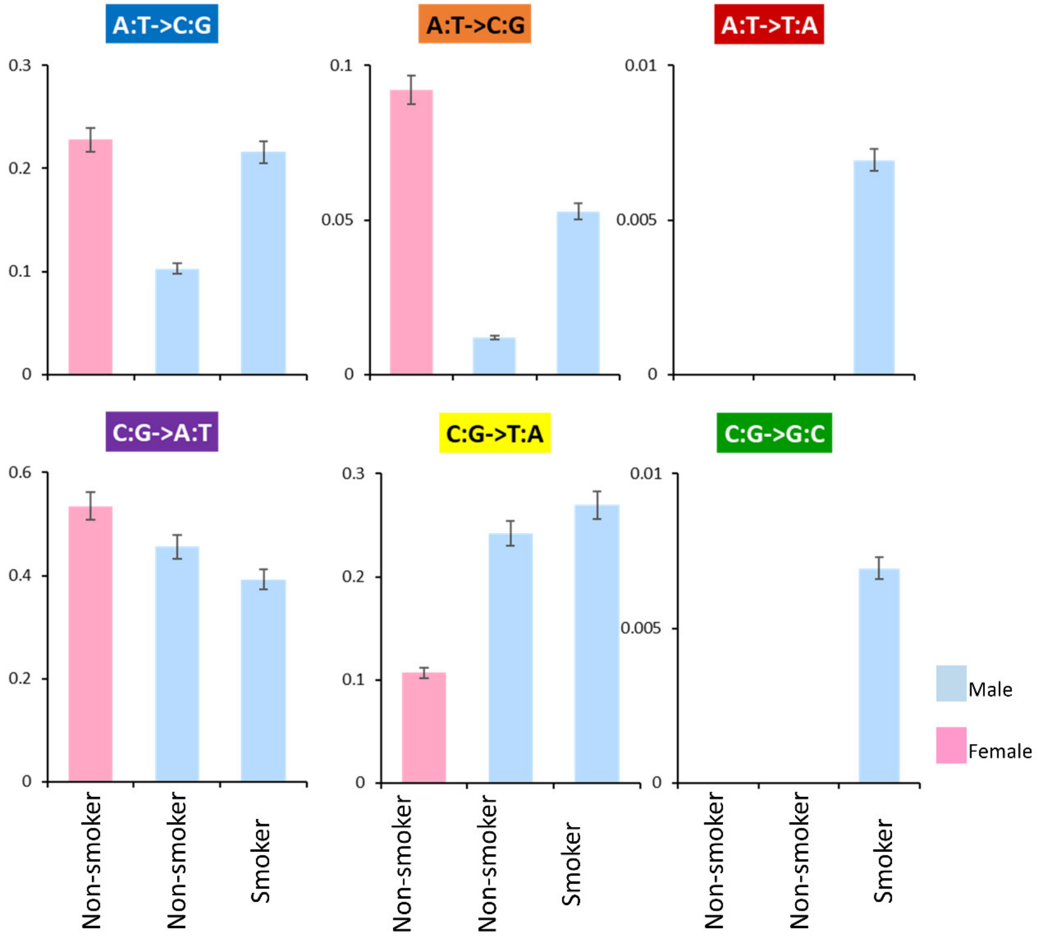

Figure S15: Gender wise nucleotide substitution profiles and mutation distribution among smoker and nonsmoker patients: y-axis is average number of nucleotide substitutions within the subgroup. In current study, only one female had smoking history and no nucleotide substitution was observed in this patient.

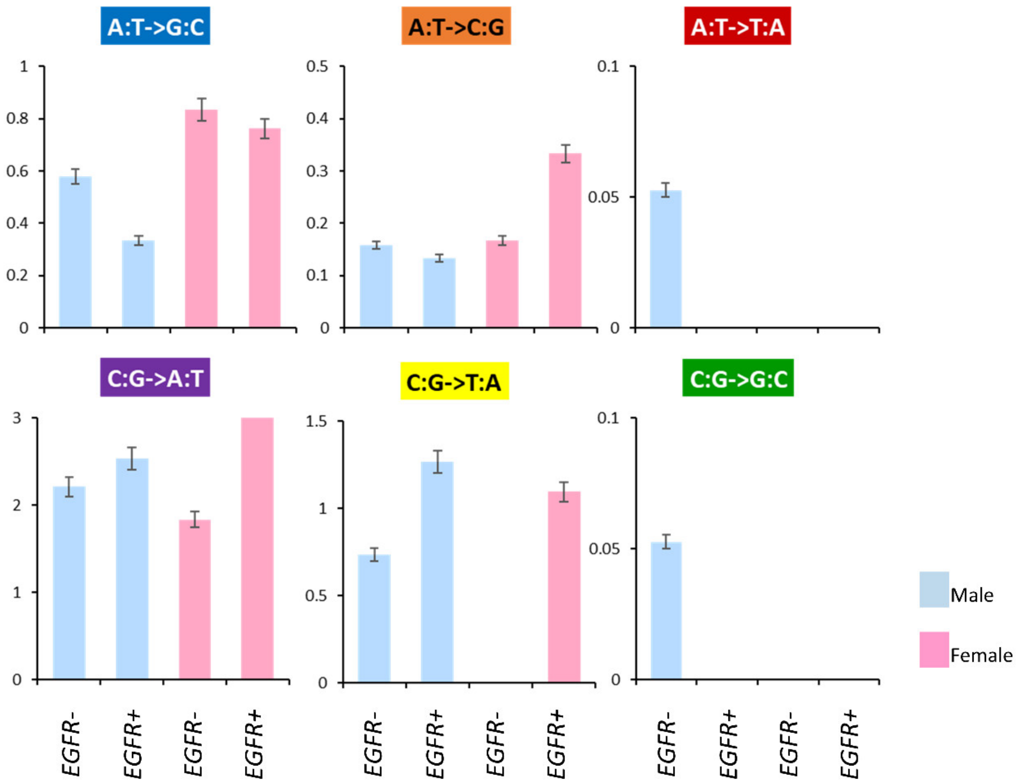

Figure S16: Gender wise nucleotide substitution profiles among patients with or without *EGFR* activating mutations (*EGFR*+ and *EGFR*- respectively): y-axis is average number of nucleotide substitutions within the subgroup.

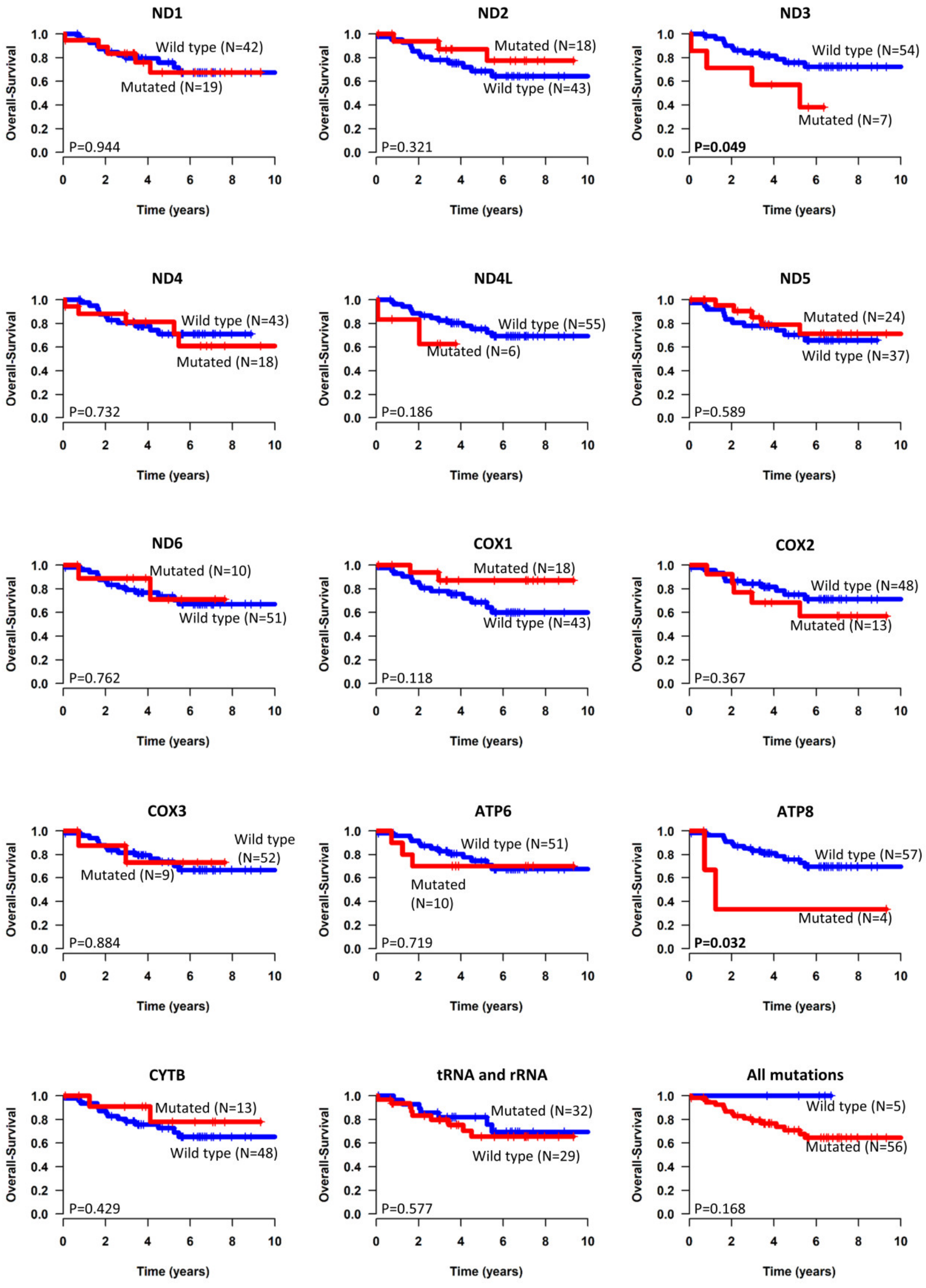

Figure S17: Kaplan-Meier estimation of overall survival of stage-I lung adenocarcinoma patients for mutations in individual mitochondrial genes and regions.

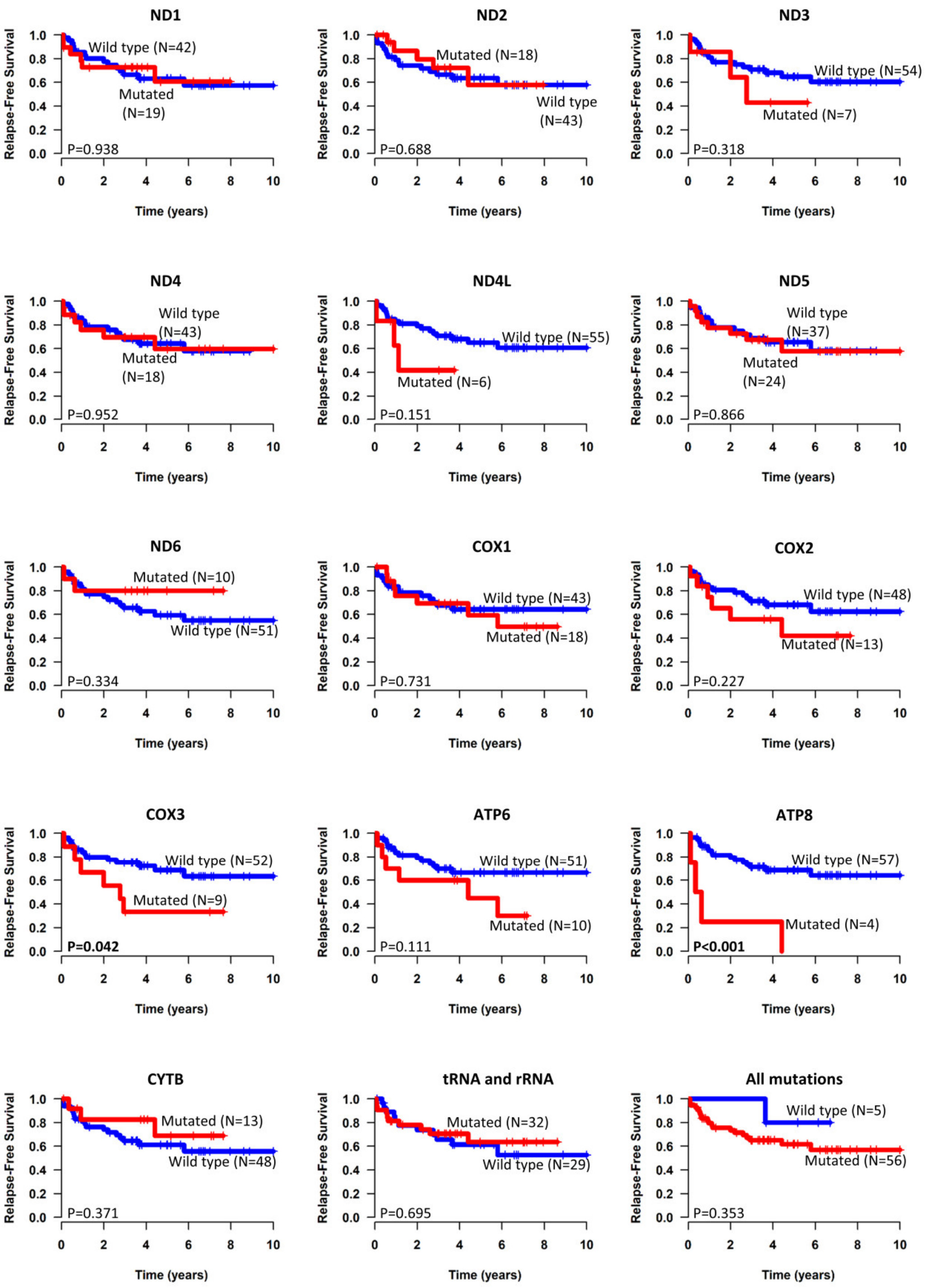

Figure S18: Kaplan-Meier estimation of Relapse-free survival of early stage lung adenocarcinoma patients for mutations in individual mitochondrial genes.

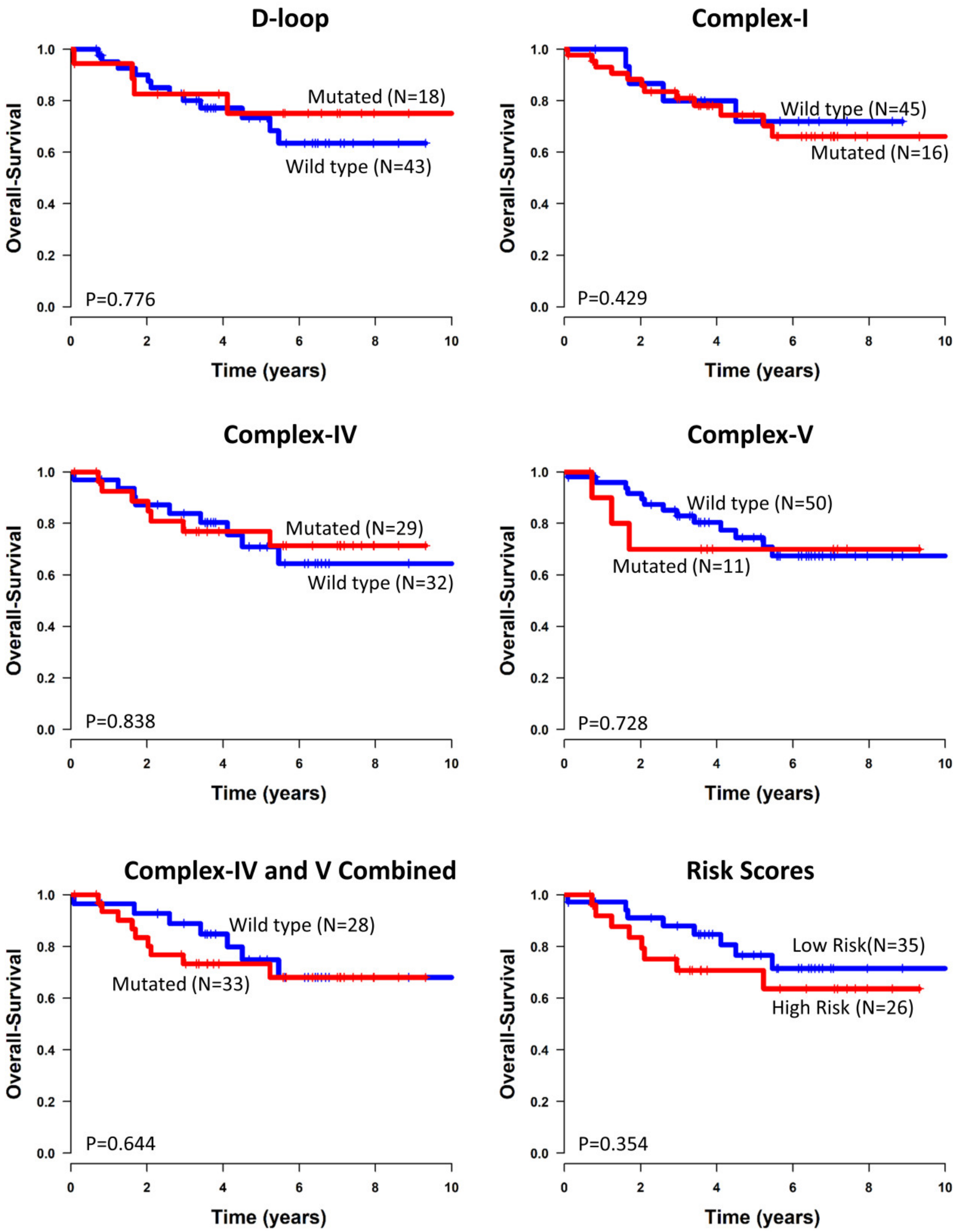

Figure S19: Kaplan-Meier estimation of overall survival of early stage lung adenocarcinoma patients for mutations in D-loop region and mitochondrial encoded OXPHOS complex genes.

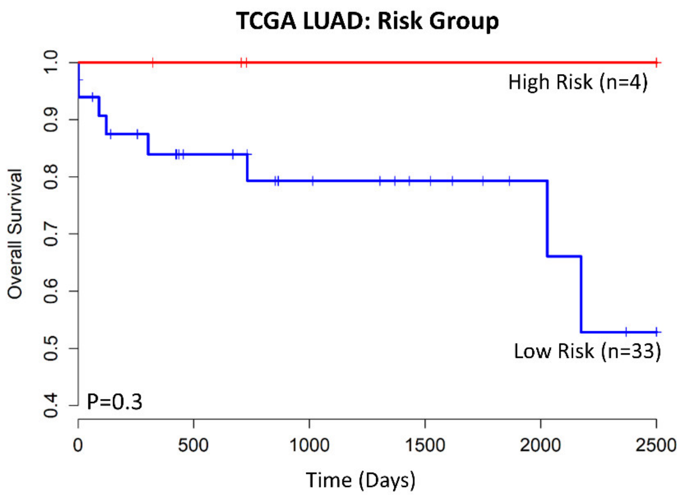

Figure S20: Kaplan-Meier estimation of overall survival for LUAD_TCGA cohort.
